# NAXD Deficiency: Heterogeneous Phenotypes and Positive Response to Niacin Treatment

**DOI:** 10.1002/jimd.70217

**Published:** 2026-07-12

**Authors:** Najmesadat Seyedkatouli, Liana N. Semcesen, Lucia Gallucci, Tim Sikora, Jean‐François Conrotte, Mei R. M. Du, Marat Kasakin, Gezime Seferi, Licia Corona, Martin Jakubec, Brunda Nijagal, Sajel Lala, Rebecca D. Ganetzky, Ana Maria Rodriguez Barreto, Marina Szlago, Melanie Wong, Margit Shah, James Nurse, Nicola Foulds, Shankar Sadagopan, Ha Nguyen Thu, Dung Vu Chi, Khanh Nguyen Ngoc, Michelle G. de Silva, Mirana Ramialison, Fernando Rossello, MitoMDT Diagnostic Network for Genomics and Omics, Shanti Balasubramaniam, Megan Ball, John Christodoulou, David Coman, Alison Compton, Ryan Davis, Lucas Dejong, Yoni Elbaum, Aleksandra Filipovska, Luke Formosa, Roula Ghaoui, Madeleine Harris, Daniella Hock, Nicole Lake, Daniel MacArthur, Pauline McGrath, Elle Martin, Sean Murray, Rocio Rius, Mike Ryan, Amanda Samarasinghe, Liana Semcesen, Stefan Siira, Tegan Stait, Diana Stojanovski, David Stroud, David Thorburn, Aimee Woods, David R. Thorburn, Matthew Lynch, Pauline McGrath, David A. Stroud, John Christodoulou, Carole L. Linster, Nicole J. Van Bergen

**Affiliations:** ^1^ Enzymology and Metabolism Group, Luxembourg Centre for Systems Biomedicine, University of Luxembourg Esch‐sur‐Alzette Luxembourg; ^2^ Department of Biochemistry and Pharmacology Bio21 Molecular Science and Biotechnology Institute, the University of Melbourne Parkville Victoria Australia; ^3^ Murdoch Children's Research Institute, Royal Children's Hospital Parkville Victoria Australia; ^4^ Metabolomics Australia, Bio21 Institute, the University of Melbourne Melbourne Victoria Australia; ^5^ Division of Clinical Genetics Nickelaus Children's Health System Coral Gables Florida USA; ^6^ Mitochondrial Medicine Frontier Program, Division of Human Genetics, Department of Pediatrics Children's Hospital of Philadelphia Pennsylvania USA; ^7^ Hospital de Niños Dr. Ricardo Gutiérrez City of Buenos Aires Argentina; ^8^ The Children's Hospital at Westmead Westmead Australia; ^9^ Faculty of Medicine and Health, University of Sydney Sydney New South Wales Australia; ^10^ University Hospital Southampton NHS Foundation Trust Southampton UK; ^11^ Faculty of Medicine, University of Southampton Southampton UK; ^12^ Center for Endocrinology, Metabolism, Genetics/Genomics and Molecular Therapy, Vietnam National Children's Hospital Vietnam; ^13^ Victorian Clinical Genetics Services, Murdoch Children's Research Institute Melbourne Victoria Australia; ^14^ Department of Paediatrics Faculty of Medicine, Dentistry and Health Sciences, University of Melbourne Melbourne Victoria Australia; ^15^ Neurosciences Unit, Queensland Children's Hospital Brisbane Australia

**Keywords:** mitochondria, NAXD, neurodegeneration, niacin, paediatric, PEBEL2

## Abstract

Early‐onset progressive encephalopathy with brain edema and/or leukoencephalopathy‐2 (PEBEL2) is a rare autosomal recessive neurometabolic disorder caused by pathogenic variants in *NAXD*, in which febrile illness or infection triggers rapid clinical deterioration. We describe nine new cases that expand the clinical and molecular spectrum. Four children showed the typical presentation of severe neurological decline following fever or illness and carried variants affecting the enzyme domain. Four cases presented with illness‐triggered cardiac dysfunction associated with variants in the mitochondrial targeting sequence. One case showed severe prenatal neurodegeneration resulting in stillbirth. In two patients, disease onset followed COVID‐19 infection. Functional analysis of five missense variants demonstrated impaired NAXD protein solubility, reduced NADHX dehydratase activity and/or decreased thermostability. Patient fibroblasts confirmed accumulation of damaged cofactors (S‐, R‐ and cyclic NADHX) and reduced NAXD protein levels. Comparative proteomic analysis revealed distinct molecular profiles in atypical cardiac and prenatal cases compared with typical neurological presentations. Four patients received high‐dose niacin (vitamin B3) and survived repeated febrile episodes. These findings support early recognition and suggest that niacin therapy may improve outcomes across the clinical spectrum of PEBEL2.

## Graphical abstract

NAXD deficiency (PEBEL2): expanding phenotypic spectrum and response to niacin therapy. PEBEL2 is a rare autosomal recessive neurometabolic disorder caused by biallelic pathogenic variants in NAXD. Analysis of nine newly reported cases reveals three distinct clinical presentations: (i) a neurological phenotype characterised by severe deterioration following febrile illness, (ii) an illness‐triggered cardiac phenotype with acute cardiac dysfunction, and (iii) a severe prenatal neurodegenerative phenotype resulting in stillbirth. Physiological stress associated with infection or fever increases metabolic demand, leading to accumulation of damaged NAD(P)H cofactors (S‐NAD(P)HX), cellular energy failure, and rapid clinical decline. Treatment with niacin (vitamin B3) was associated with survival through subsequent illness episodes and clinical improvement in four treated patients, highlighting the importance of early diagnosis and prompt initiation of niacin therapy across the PEBEL2 spectrum.

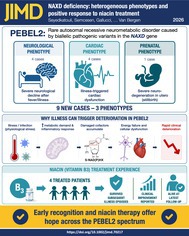

**Abbreviations**
ACMG: American College of Medical Genetics and Genomics
MTS: mitochondrial targeting signal
NAXD: NAD(P)HX dehydratase
NAXE: NAD(P)HX epimerase
OXPHOS: oxidative phosphorylation
PEBEL1: progressive early‐onset encephalopathy with brain edema and/or leukoencephalopathy‐1
PEBEL2: progressive early‐onset encephalopathy with brain edema and/or leukoencephalopathy‐2
RCA: relative complex abundance

## 1 Introduction

Progressive early‐onset encephalopathy with brain edema and/or leukoencephalopathy‐2 (PEBEL2, or NAXD disorder) is a rare, severe, lethal paediatric neurodegenerative disorder [1]. PEBEL2 is often provoked by intercurrent febrile illnesses, caused by biallelic loss of function variants in *NAXD*, encoding a repair enzyme that is enriched in the mitochondria [2, 3, 4]. The central metabolic cofactors NADH and NADPH can be converted into ‘damaged’ forms (S‐, R‐ and cyclic NAD(P)HX) by spontaneous or enzymatically catalysed hydration, and this conversion is exacerbated under stress [2, 3, 5]. The NAD(P)HX derivatives are redox inactive products that can interfere with dehydrogenase activities. Together, NAXD and the partner enzyme NAXE convert these damaged compounds back to the functional NAD(P)H cofactors [3].

There is an emerging genotype–phenotype correlation for PEBEL2 where the location of the pathogenic variant in the protein influences the clinical phenotype [1, 6, 7, 8, 9]. Pathogenic variants in the mitochondrial targeting signal (MTS) of *NAXD* (‘Mito NAXD’) were previously associated with myopathic and cardiac presentations, although only a handful of cases have been reported. Translation initiation at a downstream start codon (Met3) generates the cytoplasmic NAXD form (Cyto NAXD); additionally, alternative splicing can lead to expression of a NAXD form targeted to the ER via usage of Met2 [1, 4]. Pathogenic variants affecting both the cytoplasmic and mitochondrial forms of NAXD associate with the more common presentation of a neurological, neuroinflammatory, seizure and skin lesion phenotype. The consequence of NAXD deficiency is an accumulation of damaged cofactors, collectively termed NAD(P)HX. These inhibit dehydrogenase enzymes reliant on NAD(P)H [2], which has profound impacts on mitochondrial energy metabolism [1] and core metabolic pathways such as serine metabolism [5].

The majority of reported individuals with PEBEL2 have died after a mild illness [1, 6, 10, 11] or injury [12], except for a small number of patients who received niacin (also referred to as vitamin B3 or nicotinic acid) therapy early in their clinical presentation [7, 13]. Niacin also shows some success in preventing or slowing the progression of the related disorder, PEBEL1 [14, 15, 16, 17, 18]. However, the therapeutic efficacy of niacin for PEBEL1 and PEBEL2 may be transitory, or even toxic at extremely high doses [19, 20]. The only other anecdotal option to mitigate rapid demise of patients is the careful management of fever and illness to prevent acute decompensation. This strategy is advised for siblings with biallelic *NAXD* pathogenic variants who are not yet clinically affected, since there are no proven treatments for PEBEL2.

In this report we present nine new cases of PEBEL2. Four individuals presented with a neurological condition, two of which were triggered after COVID‐19 infection. We also describe four cases with cardiac presentation and variants affecting the MTS, and one case who was stillborn and had severe in utero neurological degeneration. Our functional genomic characterisation provides supportive evidence for *NAXD* variant pathogenicity including NAD(P)HX metabolomic assessment, protein stability, enzyme kinetic studies of novel missense variants in *NAXD*, quantitative proteomics, as well as pathway analysis from patient fibroblasts. Promisingly, four of these PEBEL2 cases have been treated with niacin, and at the current time all of these patients are still alive and have survived subsequent illnesses, further highlighting the potential for niacin‐based therapy in PEBEL2.

## 2 Methods

### 2.1 Ethics Approval and Consent to Participate

All patient cell lines used in this study were obtained following ethical approval at the Royal Children's Hospital, Melbourne (HREC/67401/RCHM‐2020 and HREC/89419/RCHM‐2022). Written informed consent was obtained from carers or parents on behalf of the patients according to the Declaration of Helsinki and has been approved by the ethical committee of the institution.

### 2.2 Fibroblast Culture

Primary cultures of fibroblasts from cases and age‐matched paediatric controls were established from skin biopsies and cells were maintained as outlined in Supporting Information.

### 2.3 NAD(P)HX Metabolite Analysis in Fibroblasts

Two extraction protocols and LC–MS systems were used for the analysis of intracellular metabolites from cultured fibroblasts in two laboratories. Case 1 intracellular metabolites were assessed using a standardised extraction protocol established by Metabolomics Australia. Detailed methodology for Case 1 is provided in Supporting Information. LC–MS measurements for Case 5 and Case 9 were performed by the Luxembourg Centre for Systems Biomedicine (LCSB) Metabolomics and Lipidomics Platform.

For Case 5, intracellular metabolite levels were assessed through an optimised extraction procedure and measured on a system consisting of an Exion LC linked to a 7500 Triple Quad MS (SCIEX) equipped with an Optiflow Pro Ion Source, which ensured higher sensitivity in metabolite detection (a detailed method description is provided in Supporting Information).

For Case 9, metabolite extraction and analysis were performed as previously described on an Agilent 1290 LC coupled to an Agilent 6560 Q‐TOF MS equipped with a Dual Agilent Jet Stream ESI source [5].

Briefly, for Cases 5 and 9, fibroblasts were cultured in 6‐well plates until approximately 90% confluence was reached. Metabolites were extracted by washing cells, adding 500–750 μL of ice‐cold extraction solution 4:1 Methanol:20 mM Tris buffer pH 8 containing thionicotinamide adenine dinucleotide (thio‐NAD) as internal standard to quench the cells, and performing phase separation by adding chloroform and 20 mM Tris buffer pH 8 to achieve a biphasic system (Chloroform/Methanol/Tris buffer = 1/1/0.9 (v/v/v)), followed by centrifugation for 5 min at 4°C and 21 000 × *g*. The polar phase was collected, snap‐frozen, and lyophilized for LC–MS analysis, while protein pellets were processed for quantification using the BCA assay.

Lyophilized extracts were reconstituted in 100 μL of 50 mM ammonium acetate and analysed by RP‐LCMS (reversed phase liquid chromatography mass spectrometry). Chromatography separation was achieved using a Polaris 3 C18‐A column (3 μm, 180 Å, 3.0 × 150 mm; Agilent) equipped with a SecurityGuard ULTRA C18 precolumn (for 3.0 mm ID columns; Phenomenex), at a constant flow rate of 300 μL/min in either gradient mode (when coupled to Q‐TOF MS) or isocratic mode (when coupled to Triple Quad MS), where solvent A was 50 mM ammonium acetate and solvent B was 100% acetonitrile (ACN). On the Triple Quad MS, a scheduled multiple reaction monitoring method in positive ion mode was performed. Target metabolites were identified by comparison of retention time, specific *m*/*z* transition and ion ratios against chemically pure standards of S‐NADHX and R‐NADHX, synthesised in‐house as previously described [2]. Details on source parameters, extracted ions and retention time for the Triple Quad method are given in Table S1.

Following manual peak curation, the peak area of each target compound was normalised to the peak area of the internal standard (thio‐NAD) and further corrected by total protein content. The statistical analyses (Student's *t*‐test) were conducted using GraphPad Prism software (V10.4.2).

### 2.4 Bacterial Expression and Purification of Wild‐Type and Mutant NAXD Proteins

Wild‐type and missense variant [p.(Pro103Leu), p.(Ser235Phe), p.(Arg250His), p.(Leu294Pro)] forms of the cytosolic NAXD isoform (cytoNAXD) were recombinantly expressed and purified as described previously [1]. The S‐NADHX substrate was synthesized as previously [2] and NAXD enzyme kinetic properties determined using a spectrophotometric assay [3]. For highly unstable missense variants, extraction, purification and enzyme assays were performed on the same day to obtain reproducible results.

NADHX dehydratase activity assays and thermostability testing were performed as described [1].

Briefly, NADHX dehydratase activity was assayed spectrophotometrically by monitoring S‐NADHX consumption at 290 nm and 37°C. A reaction mixture (total assay volume of 200 μL) containing 25 mM HEPES, pH 7.1, 2 mM MgCl_2_, 1 mM ATP, and various concentrations of S‐NADHX (0–50 μM) was pre‐incubated in UV‐Star flat‐bottom 96‐well plates in the plate reader until the signal was stable. The reaction was started by recombinant enzyme addition at a final concentration of 1.1–4 μg/mL in the reaction mixtures. Assays performed in the absence of the S‐NADHX substrate were used for background correction. For thermostability testing, purified desalted NAXD preparations (pH 7.4) were incubated at protein concentrations of 0.15–0.48 mg/mL at different temperatures (30°C–47°C) for 30 min prior to addition to the reaction mixture for enzyme activity measurement (final enzyme concentration of 2.1–8 μg/mL). All measurements were performed in at least three independent replicates and kinetic parameters were derived by fitting the data to the Michaelis–Menten equation using GraphPad Prism (V10.3).

### 2.5 Western Blot Analysis of Bacterial‐Expressed Wild‐Type and Mutant NAXD Proteins

Western blotting was performed following the protocol described [5]. Equal amounts of protein from each fraction collected during the purification process were separated by SDS–PAGE and transferred onto a membrane. NAXD protein bands were detected using a primary antibody against NAXD (anti‐NAXD, cat. no. HPA010551; Sigma‐Aldrich, Merck; 1:200) followed by an IRDye‐conjugated secondary antibody (goat anti‐rabbit IgG, IRDye 800CW, cat. no. 926‐32 211; LI‐COR; RRID: AB_621843; 1:5000).

### 2.6 Mammalian Expression and Purification of Wild‐Type and Mutant NAXD Proteins and Western Analysis

CytoNAXD was cloned into a mammalian expression vector with an N‐terminal FLAG tag, then the missense variants p.Ile149Ser, p.Ser235Phe, p.Arg250His and p.Leu294Pro were introduced by site‐directed mutagenesis (primers in Table S6). Protein was overexpressed in HEK293T cells for subcellular fractionation and Western blot studies. Full methods and plasmid maps are in Supporting Information.

### 2.7 p.Met1? Subcellular Analysis Studies

MitoNAXD was cloned into a mammalian expression vector with a C‐terminal FLAG tag, then the p.Met1? variant was introduced by site‐directed mutagenesis. Protein was overexpressed in either HEK293T cells for subcellular fractionation and Western blot studies or COS7 cells for intracellular localisation studies. Full methods and plasmid maps are in Supporting Information.

### 2.8 Quantitative Proteomics in Patient Fibroblasts

Sample preparation and data acquisition for proteomic analysis of proband fibroblasts were performed independently against batch controls for each case, with samples prepared as previously described [21]. Fibroblasts from each proband were analysed in triplicate, while fibroblasts from five unrelated paediatric controls were analysed without technical replicates. Peptides were analysed by liquid chromatography–tandem mass spectrometry (LC–MS/MS) acquired by data‐independent acquisition (DIA). For Case 1 and Case 9, LC–MS/MS was carried out on an Orbitrap Ascend Mass Spectrometer (Thermo Scientific) equipped with an Ultimate 3000 HPLC (Thermo Scientific), an Acclaim Pepmap nano‐trap column (Dionex‐C18, 75 μm × 2 cm) and an Acclaim Pepmap RSLC analytical column (Dionex‐C18, 75 μm × 50 cm). Peptides were separated across a 95‐min gradient using a previously described method [21]. For Case 5, LC–MS/MS was carried out on an Orbitrap Astral mass spectrometer (Thermo Scientific) equipped with a Vanquish Neo UHPLC (Thermo Scientific) and using the heated trap and elute setup, an Acclaim Pepmap nano‐trap column (Dionex—C18, 100 Å, 75 μm × 2 cm) and a high‐throughput μPAC Neo analytical column (Thermo Scientific, 5.5 cm). Peptides were separated across a 30‐min gradient using a previously described method [22].

Raw files were searched using Spectronaut (version 18.6.231227.55695 for Case 9 and 19.0.240606.62635 for Cases 1 and 5, Biognosys) and searched against the human UniProt database (42 386 entries) in a direct DIA+ (deep) workflow. Default BSG Factory Settings were used with the following modifications: ‘Exclude Single Hit Proteins’ was selected, and ‘Major Group Top N’ and ‘Minor Group Top N’ were deselected. For Case 5, the same search settings outlined above were used, in addition to the ‘Precursor PEP Cutoff’ adjusted to 0.05, and both ‘Protein Qvalue Cutoff (Run)’ and ‘Protein PEP Cutoff’ adjusted to 0.01, to account for a more sensitive mass spectrometer used. Raw data was imported into Perseus (v.1.6.15.0) [23], and MS2 quantities were log2 transformed and filtered for at least 2 valid values in either the proband or control groups. A two‐sided *t*‐test was performed and visualised by a volcano plot using the scatter plot function. Mitochondrial proteins were annotated using the MitoCarta3.0 database [24], and significant thresholds were set to *p* value = 0.05 (−log10 = 1.301) and fold‐change = +/−1.5 (log2 = +/−0.585). Range plots for residual NAXD abundance were calculated from the MS2 quantity of NAXD in the proband relative to the median control value, as previously described [21]. Peptide heatmaps were generated by exporting the MS2 quantity of peptides from Spectronaut, and their log2 transformed values were visualised in GraphPad Prism (10.4.1). The Relative Complex Abundance (RCA) analysis was conducted using a previously described in‐house script [21], and visualised in R (version 4.3.0) and Rstudio (2024.04.2 + 764).

### 2.9 Bioinformatic Analysis of Proteomics Data

Differential protein expression analysis was conducted in R (version 4.1.2) with packages DEqMS (version 1.12.1) [25] and limma (version 3.50.3) [26]. Normalisation was first performed by median centring. Correlation between replicates of the same sample was estimated with limma's duplicateCorrelation function and incorporated into a linear model, before using empirical Bayes moderation of *t*‐statistics [27]. DEqMS' spectraCounteBayes function was used to further adjust the *t*‐statistics by accounting for variance according to the minimum number of peptides used per protein for quantification. *p* values were adjusted by the Benjamini‐Hochberg method [28] and an adjusted *p* value threshold of 0.05 identified significant differentially expressed proteins for the contrast of interest. Over‐representation analysis was then performed to identify Reactome [29] pathways over‐represented in these significant gene sets, using clusterProfiler (version 4.2.2) [30, 31] and gprofiler2 (version 0.2.3) [32, 33].

## 3 Results

### 3.1 Clinical and Genetic Description of NAXD Cases

**FIGURE 1:**
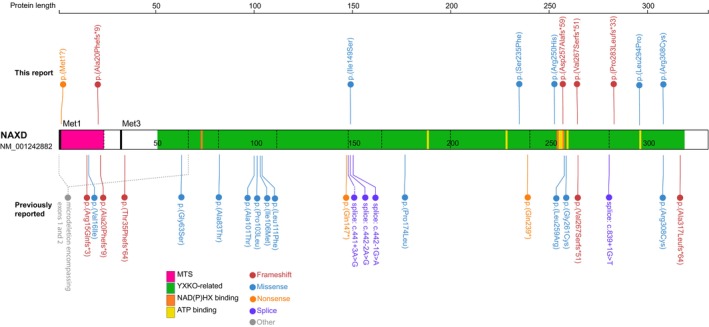
*NAXD v*ariant location. Location of variants described in this report are shown above the NAXD structure, in reference to NAXD transcript NM_001242882.1 (isoform c). Variants previously reported are shown at the bottom. Image was generated with ProteinPaint [34]. YXKO‐related; ATP‐dependent (S)‐NAD(P)H‐hydrate dehydratase domain of NAXD. MTS; mitochondrial targeting signal.

Here we describe 9 new cases with pathogenic variants in *NAXD* and varied clinical presentations (Table 1), harbouring novel and previously reported *NAXD* variants (Tables 2 and S2, Figure 1). Full clinical case summaries including niacin and supplement information and detailed genetic reports are available in Supporting Information, and American College of Medical Genetics and Genomics (ACMG) classifications in Table S3.

**TABLE 1:** Clinical summary of NAXD patients.

Case number	Case 1	Case 2	Case 3	Case 4	Case 5	Case 6	Case 7	Case 8	Case 9	Previously reported cases^a^	
Prediction for NAXD variants	Combined NAXD	Combined NAXD	Combined NAXD	Combined NAXD	Combined NAXD and Mito NAXD	Mito NAXD	Mito NAXD	Mito NAXD	Combined NAXD	Mito NAXD^b^ (*n* = 5)	Combined NAXD (*n* = 11)
Gender	Male	Male	Male	Female	Female	Male	Female	Male	Male	3M, 2F	7M, 4F
Infections prior to presentation	Yes	Yes	Yes	Yes		Yes, gastrointestinal and enteritis	NA	NA	NA	3/5 2 not reported	4/9 2 not reported
Fever	Yes	Yes	Yes	Yes	Yes	Yes	NA	NA	NA	5/5	8/11
Current status	Alive	Died	Alive	Alive	Alive	Died	Died (4 yo)	Died (5 yo)	In utero demise	1/5 alive	3/10 alive
Neurodegeneration	Yes	Yes	No	Yes	No	NA	NA	NA	Yes	1/5	9/10 1 not reported
Skin and/or mucosal lesions	Yes	Yes	No	No	No	No	NA	NA	NA	0/5	9/11
Movement abnormality and/or hypotonia	Yes	Yes	Yes	No	No	No	NA	NA	NA	3/3 2 not reported	7/9 2 not reported
Cardiac presentation	No	No	No	No	Yes	Yes, myocarditis	Yes, suspected myocarditis	Yes, myocarditis	NA	3/5	2/10 1 not reported
Niacin treatment	Yes	No	Yes	Yes	Yes	No	No	No	NA	Reported for 1 individual	Reported for 4 individuals
Niacin regime^c^	Nasogastric 10 mg/ml at 8 months Ongoing; 50 mg daily	NA	Initially 200 mg (100 mg twice a day) at 8 months then increased to 300 mg/day	40 mg/day bolus dose	Nicotinamide 15 mg/kg/day in 2 doses	NA	NA	NA	NA	NA	NA

Abbreviation: NA, information not available.

^a^ For full clinical information about previously published cases, see Table S2.

^b^ One case was reported with two variants: one Mito NAXD and one Combined NAXD.

^c^ For full information on niacin and other supplement treatment details refer to Supporting Information which contains full clinical reports.

**TABLE 2:** In silico analysis of NAXD variants.

	GnomAD population frequence (MAF)		Mito or Combined NAXD classification? [1]	Genebank transcript ID	NM_001242882.1^b^	NM_018210.3^c^
				Genebank protein ID	NP_001229811	NP_060680
	Heterozygotes	Homozygotes				
				Relative expression (based on EST) [2]	81%	0%
Case 1	14	0	Combined NAXD	cDNA position	c.749G>A	c.803G>A
				AA change	p.(Arg250His)	p.(Arg268His)
				Consequence^a^	Missense. Pathogenic (II)	
	1	0	Combined NAXD	cDNA position	c.770del	c.824del
				AA change	p.(Asp257Alafs*59)	p.(Asp275Alafs*29)
				Consequence^a^	Frameshift. Pathogenic (Ia)	
Cases 2 and 3	19	0	Combined NAXD	cDNA position	c.922C>T	c.1112C>T
				AA change	p.(Arg308Cys)	p.(Ser371Leu)
				Consequence^a^	Missense. Reduced NADHX dehydratase activity [3]. Pathogenic (II)	
	0	0	Combined NAXD	cDNA position	c.794_798dup	c.848_852dup
				AA change	p.(Val267Serfs*51)	p.(Val285Serfs*21)
				Consequence^a^	Frameshift. Pathogenic (Ia)	
Case 4	1	1	Combined NAXD	cDNA position	c.446T>C	c.500T>C
				AA change	p.(Ile149Ser)	p.(Ile167Ser)
				Consequence^a^	Missense. Likely pathogenic (IV)	
Case 5	0	0	Combined NAXD	cDNA position	c.881T>C	c.1071T>C
				AA change	p.(Leu294Pro)	p.(=)
				Consequence^a^	Missense. Pathogenic (IIIa)	
	12	0	Mito NAXD	cDNA position	c.51_54delAGAA	c.105_108delAGAA
				AA change	p.(Ala20Phefs*9)	p.(Ala38Phefs*9)
				Consequence^a^	Frameshift. Loss of mitochondrial targeting signal (MTS). Expression of cNAXD at Met3, experimentally verified ^4^. Previously reported ^3^. Pathogenic (Ia)	
Cases 6, 7 and 8	3	0	Mito NAXD	cDNA position	c.1A>T	Untranslated region
				AA change	p.Met1?	N/A
				Consequence^a^	Start loss of Met1. Predicted expression of cNAXD at Met3. Pathogenic (Ia)	
Case 9	1	0	Combined NAXD	cDNA position	c.704C>T	c.758C>T
				AA change	p.(Ser235Phe)	p.(Ser253Phe)
				Consequence^a^	Missense. Pathogenic (II)	
	0	0	Combined NAXD	cDNA position	c.848del	c.1038del
				AA change	p.(Pro283Leufs*33)	p.(Ser347fs)
				Consequence^a^	Frameshift. Pathogenic (Ia)	

Abbreviation: n/a, not available.

^a^ See Table S3 for full ACMG pathogenicity scoring based on NP_001229811.

^b^ This variant (3) differs in the 5′ UTR and 5′ coding region, uses an alternate start codon, and uses an alternate splice site that causes a frameshift in the 3′ coding region, compared to variant 1. The encoded isoform (c) has distinct N‐ and C‐termini and is shorter than isoform a.

^c^ This variant (1) represents the longest transcript and encodes the longest isoform (a).

The location of the pathogenic variant is predicted to associate with the clinical phenotype [9], whereby variants affecting both the Cyto NAXD and Mito NAXD activity (Combined NAXD) primarily associate with neurodegeneration and skin lesions, whilst variants in the MTS of *NAXD* (MitoNAXD) primarily associate with a cardiac/muscular phenotype. Four cases showed the more ‘typical’ PEBEL2 presentation (Cases 1, 2, 3 and 4), where a rapid neurometabolic decline was triggered after an otherwise trivial childhood illness, and these variants were predicted to lead to Combined NAXD deficiency. Four cases (Cases 5–8) presented with a cardiac phenotype, where one of the two variants in Case 5 (p.(Ala20Phefs*9)), and the homozygous p.Met1? variant in Cases 6, 7 and 8, were predicted to lead to MitoNAXD deficiency. The family pedigree is in Figure S1 and a normal brain MRI for Case 6 is in Figure S2. Lastly, Case 9 presented with severe neurological damage in utero (Figure S3) and was delivered stillborn with variants classified as ‘Combined NAXD’. Fibroblast cell lines from skin biopsies were available from Cases 1, 5 and 9 for functional studies.

### 3.2 NAD(P)HX Accumulation in Fibroblasts

**FIGURE 2:**
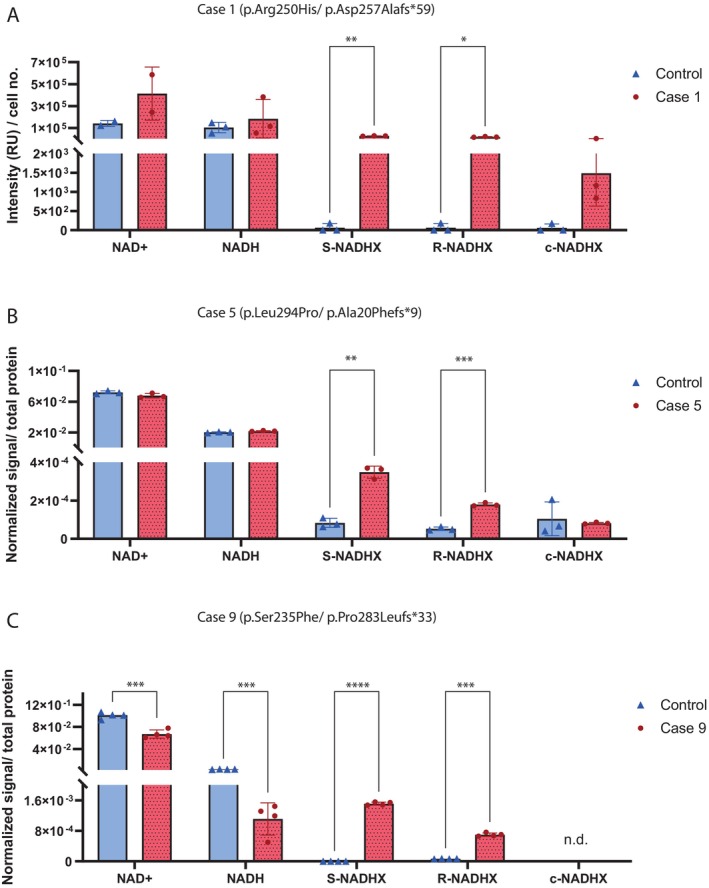
NAD(P)HX accumulation in NAXD patient fibroblasts. NAD(P)HX levels were measured in cell extracts after cultivation of fibroblasts from (A) Case 1, (B) Case 5 and (C) Case 9, demonstrating a clear accumulation of both S‐ and R‐NADHX in patient fibroblasts compared to a paediatric control. c‐NADHX levels in Case 9 were below the limit of detection of the LC–MS system used. Note that NADHX intracellular levels were not quantifiable in control fibroblast lines used for Case 1 and 9. For analysis of Case 1, NADHX species were putatively identified, as pure standards were not available in the reference laboratory. For Case 1 peak intensity was normalised to cell number. For Cases 5 and 9, each peak area was normalised to the internal standard (level 1 identification was achieved against S‐ and R‐NADHX standards; Table S5) and total protein concentration. Data are means ± SD, *n* ≥ 3. Statistical significance was determined using an unpaired *t* test with Welch correction, significance is versus control, *p < 0.05, **p < 0.01, ***p < 0.001, ****p < 0.0001.

Intracellular levels of NAD(P)HX were measured by LC–MS using skin fibroblast extracts from Cases 1, 5 and 9. S‐NADHX and R‐NADHX levels were significantly increased in patient fibroblasts compared to paediatric control fibroblasts (Figure 2). NAD^+^ and NADH levels were unchanged in patient cells compared to healthy control cells, except for Case 9 where NAD(H) levels were moderately decreased (Figure 2C). However, it should be noted that the fibroblasts from Case 9 exhibited slower growth than the other cell lines, which complicates direct comparison of metabolite‐level changes. Nonetheless, the clear accumulation of damaged NADHX derivatives in all tested patient cell lines functionally confirms impaired NAXD repair activity in these individuals. Note that NADHX intracellular levels were below the limit of detection in the healthy control fibroblasts used for Cases 1 and 9, with the exception of R‐NADHX in the control line of Case 9. Lower fold changes were measured for S‐ and R‐NADHX in the fibroblasts from Case 5 versus healthy control fibroblasts (4.2‐fold change for S‐NADHX, 3.4‐fold change for R‐NADHX) than for Case 1 (286‐fold change for R‐NADHX) and Case 9 fibroblasts (10.2‐fold change for R‐NADHX). This may be explained by the fact that the p.(Ala20Phefs*9) allele in Case 5 (cardiac phenotype), while leading to a loss of the mitochondrial and ER forms of NAXD, allows for residual expression of active cytosolic NAXD protein [6]. For Cases 1 and 9, the compound heterozygous variants are predicted to cause a loss of activity of all the main expression products of NAXD.

### 3.3 Recombinant NAXD Enzyme Kinetics of Missense Variants

As the *NAXD* missense variants p.(Arg250His) (Case 1), p.(Leu294Pro) (Case 5), p.(Ser235Phe) (Case 9) were initially classified as VUS, we performed enzyme kinetic studies with recombinant NAXD protein to support pathogenicity. We also included the previously reported p.(Pro103Leu) variant [1] in these studies, as enzyme kinetic parameters had not been determined for that variant before, and a knock‐in mouse model harbouring the homologous point mutation is under development. The variants were introduced by site‐directed mutagenesis into human cytosolic *NAXD* cDNA, expressed as His‐tag fusion proteins in *E. coli* and purified by affinity chromatography.

**FIGURE 3:**
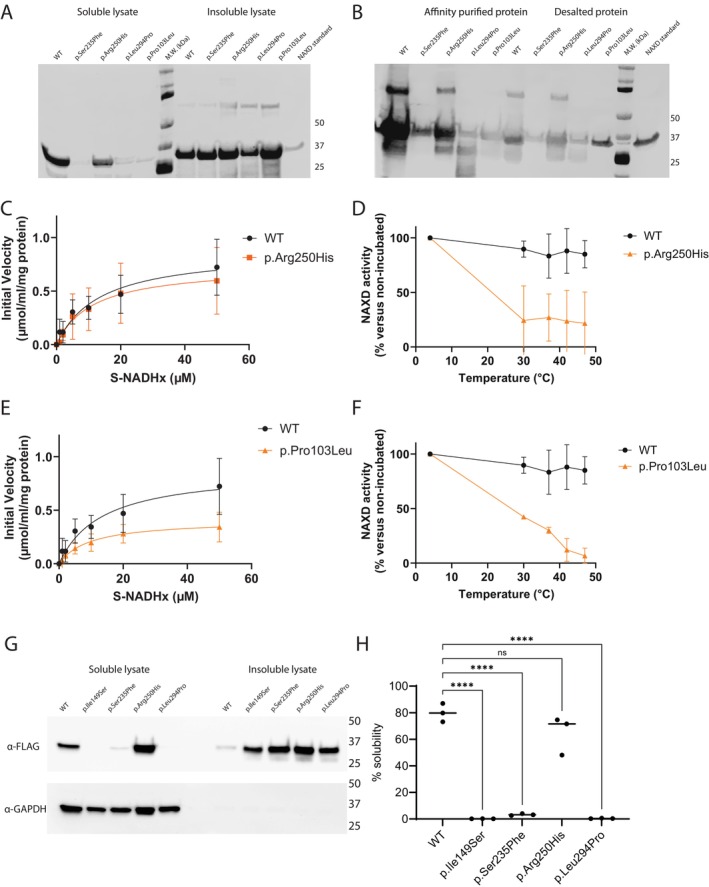
Stability and enzyme kinetic analyses of NAXD missense variants. The *NAXD* missense variants [p.(Pro103Leu), p.(Ile149Ser), p.(Ser235Phe), p.(Arg250His) or p.(Leu294Pro)] were introduced by site‐directed mutagenesis into human *NAXD* cDNA, then expressed and purified as bacterial recombinant expressed or human HEK293T expressed proteins. (A and B) Western blot analysis of different steps during extraction and purification of missense variants expressed in bacteria with anti‐human NAXD antibody (HPA010551, Sigma‐Aldrich, Merck), demonstrating insolubility or instability of NAXD protein with some NAXD variants. Enzyme activity analyses were performed for variants that yielded sufficient purified protein [p.(Arg250His) and p.(Pro103Leu)]. (C) Michaelis–Menten plot for wild type and p.(Arg250His) cytoNAXD variant (Case 1). (D) Thermostability of wild‐type (WT) and p.(Arg250His) proteins. (E) Michaelis–Menten plot for wild type and p.(Pro103Leu) cytoNAXD variant from [1]. (F) Thermostability of wild‐type (WT) and p.(Pro103Leu) proteins. Data represent means ± SD for at least 3 independent replicates. (G and H) Western blot analysis of different steps during extraction and purification of missense variants expressed in HEK293T cells with an N‐terminal FLAG tag, demonstrating insolubility or instability of NAXD protein with some NAXD variants.

To verify bacterial expression of all NAXD variants and assess potential protein loss during extraction or purification, we performed Western blotting on multiple fractions; the final affinity‐purified, desalted protein was used for enzymatic assays. Probing with anti‐human NAXD confirmed robust expression of all variants, as indicated by strong bands at the expected molecular weight in the insoluble lysate fractions (Figure 3A). In contrast, comparison of soluble fractions revealed markedly reduced solubility for p.(Ser235Phe), p.(Leu294Pro) and p.(Pro103Leu) relative to the wild‐type protein (Figure 3A). Except for p.(Arg250His), the missense variants yielded substantially lower amounts of soluble protein, consistent with decreased stability of the mutant enzymes.

For variants p.(Leu294Pro) and p.(Ser235Phe), protein loss across the purification procedure was so pronounced that the remaining material was insufficient for reliable determination of specific activity, and enzymatic activity was barely detectable (Figure S4). By contrast, purified and desalted protein from variants p.(Arg250His) and p.(Pro103Leu) was present in sufficient quantities to allow enzymatic characterisation (Figure 3B). Both variants exhibited substantial residual enzymatic activity. The p.(Arg250His) variant showed *V*_max_ and *K*_*m*_ values that were not significantly different from the wild‐type enzyme, whereas the previously reported p.(Pro103Leu) variant [1] displayed an approximately 2‐fold reduction in maximal velocity (Table S4; Figure 3C,E). However, both mutant enzymes retained less than 50% of their initial activity after pre‐incubation at 30°C or higher temperatures, indicating a pronounced loss of thermostability relative to the wild‐type protein, which remained stable up to the highest temperature tested (47°C) (Figure 3D,F).

To confirm whether NAXD missense variants were also insoluble when expressed in mammalian cells, we cloned the missense variants p.(Arg250His) (Case 1), p.(Ile149Ser) (Case 4), p.(Leu294Pro) (Case 5), and p.(Ser235Phe) (Case 9) into an N‐terminal FLAG mammalian expression vector and expressed proteins in HEK293T cells. As with the bacterially expressed missense variants, both p.(Ser235Phe) and p.(Leu294Pro) variants were insoluble, and p.(Arg250His) was soluble (Figure 3G,H). We also confirmed that the p.(Ile149Ser) variant caused protein insolubility.

### 3.4 Quantitative Proteomics

**FIGURE 4:**
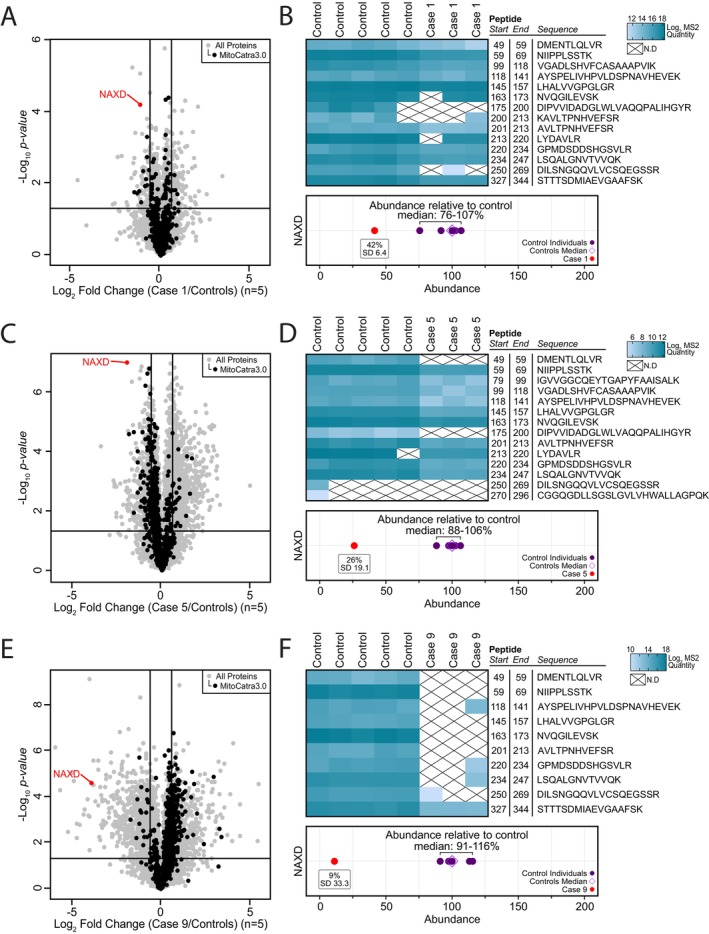
Quantitative proteomics. Volcano plot showing protein abundances of mitochondrial proteins (based on MitoCarta3.0 annotations) detected through quantitative proteomics in the fibroblasts from (A) Case 1, (C) Case 5 and (E) Case 9 relative to healthy controls (*n* = 5). NAXD was one of the most significantly decreased proteins in all cases. Across the protein sequence, the number of NAXD peptides detected was lower in (B) Case 1, (D) Case 5 and was nearly absent in (F) Case 9 compared to controls (upper panel). The residual abundance of NAXD, represented as a percentage relative to the control median, was significantly lower in all cases (lower panel).

Quantitative proteomics is a reliable tool to determine both global proteome changes, and the potential functional significance of VUS in mitochondrial disease patients [21, 35, 36]. We conducted whole‐cell quantitative proteomics on fibroblasts from Cases 1, 5 and 9, and compared the outcomes to 5 previously reported PEBEL2 patients [1, 12]. In all 3 new cases, NAXD was one of the most significantly decreased proteins, mitochondrial or otherwise (Figure 4A,C,E). In Cases 1 and 5, slightly reduced numbers of NAXD peptides were detected compared to control samples. However, only 1–4 NAXD peptides were detected in Case 9 across the length of the protein, strongly suggestive of a near absence of NAXD protein (Figure 4B,D,F, upper panel). All NAXD peptides used for protein abundance quantification were those detected across shared sequence regions between Mito NAXD and Cyto NAXD, and no unique peptides to MTS (affecting Mito NAXD) were detected in any patient or control sample, which is consistent with the MTS being processed or cleaved upon mitochondrial import. The residual abundance of NAXD protein in patient fibroblasts was quantified and measured to be reduced to 42% of control median (normal range for in batch controls = 76%–107%), 26% of control median in Case 5 (normal range for in batch controls = 88%–106%) and 9% of control median in Case 9 (normal range for in batch controls = 91%–116%) (Figure 4B,D,F, lower panel).

Since previous reports demonstrated a modest but significant reduction in mitochondrial oxidative phosphorylation (OXPHOS) and mitoribosomal proteins detected by relative complex abundance (RCA) analysis in PEBEL2 patient fibroblasts with a neurological presentation [12], we compared these previously reported cases to the proteomic profiles of Cases 1, 5 and 9. Case 1, with a neurological presentation, had a significant reduction in the RCA of complex I (92%) compared to controls, in line with previous PEBEL2 cases [12], but no changes in abundance of mitoribosome subunits. The abundance of Complex III was also significantly reduced in Case 1 (72%), which was not seen in any of the previously analysed *NAXD* cases. Previous PEBEL2 cases also had reduced complex IV and V abundance in addition to reduced complex I. Interestingly, both Case 5 with a cardiac presentation and Case 9 with severe in utero neurodegeneration had no significant decreases to either the mitochondrial OXPHOS, or the mitoribosomal proteins (Table S5 and Figure S5). In contrast, Case 5 had significantly increased mitoribosomal subunits (112%) and Case 9 had increased complex I (114%) and complex V (111%).

Proteomic datasets of Cases 5 and 9 were compared to the datasets from five previously reported PEBEL2 cases [12]. Qualitative analysis demonstrated that the vast majority of differentially expressed proteins were unique to each group. There was no overlap in all three datasets in the same direction for either individual proteins (Figure S6A) or common pathways based on over‐representation analysis (Figure S6B). Examination of the top enriched Reactome pathways for the five previously reported PEBEL2 cases [12] showed enrichment of several cell‐extracellular matrix related biological pathways, as well as gluconeogenesis in up‐regulated proteins, whilst there was an enrichment of cholesterol biosynthesis and multiple mitochondrial related pathways including TCA cycle, mitochondrial translation and pyruvate metabolism in down‐regulated proteins (Figure S7A). In contrast, for Case 5, pathways enriched in up‐regulated proteins were involved in cell cycle and DNA repair, whilst pathways enriched in down‐regulated proteins included lipid metabolism and innate immune system (Figure S7B). Case 9 had a modest enrichment in the ‘metabolism’ pathway for upregulated proteins, and extracellular matrix organisation for down‐regulated proteins (Figure S7C).

### 3.5 p.Met1? Expression and Intracellular Localisation Analysis

**FIGURE 5:**
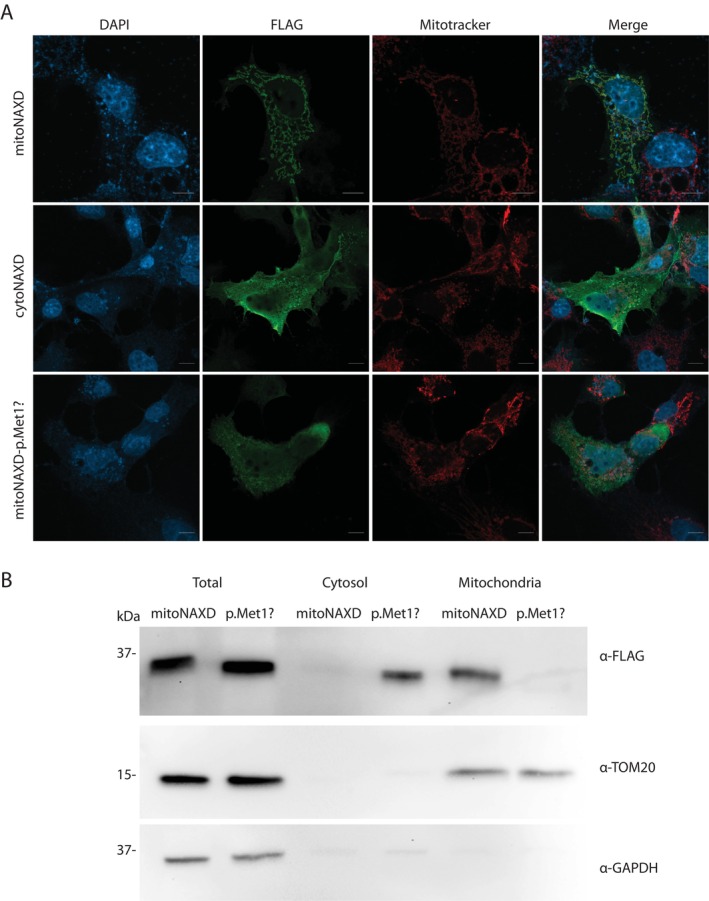
Western blot and subcellular analysis of NAXD p.Met1? variant. (A) COS7 cells were transfected with mitoNAXD‐FLAG or mitoNAXD p.Met1?‐FLAG (p.Met1?) variant (introduced by site‐directed mutagenesis), or FLAG‐cytoNAXD. Cells were counter‐stained for mitochondria (MitoTracker Red CMXRos) and nucleus (DAPI), demonstrating cytosolic localisation of p.Met1? NAXD. Scale bar = 10 μm. (B) HEK293 cells were transfected with mitoNAXD‐FLAG or mitoNAXD p.Met1?‐FLAG, then sub‐fractionated into total, cytosolic and mitochondrial fractions. The mitoNAXD protein was predominantly present in the mitochondrial fraction, with a low‐level expression in the cytosol. Conversely, the mitoNAXD p.Met1? protein was present in the cytoplasm but not the mitochondria, and the protein migrated with a slightly lower molecular weight (see Figure S9). GAPDH was used as a marker protein for the cytosol, whilst TOM20 was used as a marker protein for the mitochondria.

Cases 6, 7 and 8 all had the same homozygous start‐loss variant (c.1A>T; p.Met1?), which had not been reported previously. We predicted that the loss of p.Met1? would prevent expression of mitoNAXD but allow expression of cytoNAXD when Met3 was utilised. To determine the functional consequences of the p.Met1? variant we generated a mitoNAXD expression construct with a C‐terminal FLAG tag and introduced the patient variant (c.1A>T) by site‐directed mutagenesis. To validate intracellular location, COS7 cells were transfected with the same constructs, or FLAG‐cytoNAXD, stained with MitoTracker Red CMXRos Dye, then fixed and immunostained for the FLAG epitope. There was a clear colocalization of wild‐type mitoNAXD with the mitochondria, whilst cytoNAXD was cytosolic. The p.Met1? NAXD protein was expressed but failed to localise to mitochondria, remaining restricted to the cytoplasm (Figures 5A and S8A). Protein was overexpressed in HEK293T cells, which were then fractionated into cytosolic and crude mitochondrial fractions. Western blot analysis showed that mitoNAXD was predominantly expressed in the mitochondria, whilst p.Met1? protein was mainly confined to the cytoplasm (Figures 5B, S8B and S9). Additionally, the p.Met1? protein was slightly smaller than the wild‐type counterpart (Figures 5B, S8B and S9), suggesting initiation at Met3, without the 31 amino acid MTS being expressed.

## 4 Discussion

Pathogenic variants in *NAXD* are predominantly associated with a rapidly progressive neurologic deterioration triggered by febrile illness [1]. However, variants in the MTS of *NAXD* are increasingly being associated with a cardiac/muscular phenotype [9]. Here, we report 9 individuals with pathogenic *NAXD* variants, further expanding the clinical spectrum of PEBEL2. Four cases had a cardiac presentation and pathogenic variants in the MTS, and one child was stillborn with significant in utero neurological deterioration. This expands our understanding of the clinical spectrum due to pathogenic variants in *NAXD*, beyond the most common neurological presentation of PEBEL2.

We also consolidated the evidence base of niacin as a promising treatment for PEBEL2 [9, 16, 37] and propose that PEBEL2 should now be considered a treatable mitochondrial disease. In our report, the four individuals under niacin treatment have survived repeated periods of illness or infection that otherwise may have precipitated their demise, and all are still alive. There are now nine PEBEL2 cases (including those reported here, Table S2) that have been treated with niacin [7, 12, 13, 20, 37], and at the time of reporting all but one individual was still alive. Although long‐term niacin efficacy and safety remain to be determined, one child with PEBEL2 was reported to be on niacin (250–500 mg/day) for almost 6 years, without major side effects except a perioral rash [37], and had improvement in their neurological symptoms. Niacin also holds promise for treatment of the closely related disorder of the nicotinamide nucleotide repair system, PEBEL1, caused by biallelic loss of function pathogenic variants in *NAXE*. Eleven individuals with PEBEL1 have received niacin treatment (sometimes in conjunction with other supplements or mitochondrial cocktails), and of these, seven were reported as still alive (Table S2). Despite positive outcomes for a large proportion of patients with PEBEL1 and PEBEL2 on niacin, there are instances of concern. The oldest reported individual with PEBEL2, whose deterioration was triggered after a head injury, received niacin late in their clinical journey, which failed to prevent their demise [12]. An individual with PEBEL1 received dose‐escalation niacin therapy; however, this failed to halt their disease [19]. Evidence from *naxd−/−* zebrafish larvae demonstrated that nicotinic acid was able to substantially prolong survival and locomotion above untreated *naxd−/−* larvae, but after 20 days post fertilisation their survival decreased. Nicotinic acid supplementation increased NAD+ and NADH levels in *naxd−/−* zebrafish; however, this was accompanied by overloaded S‐ and R‐NADHX [38]. Taken together, there are clear benefits of early niacin intervention, but caution may be required to prevent NADHX overload. Additionally, a deeper understanding of the biological pathway perturbations subsequent to *NAXD* and *NAXE* deficiency may identify even more efficient and/or targeted therapies. For example, detailed metabolomic and biochemical analyses demonstrated partial reversal of some of the cellular impacts of NAXD deficiency upon nicotinamide riboside or inosine treatment [5]. Additionally, high‐throughput drug screening may identify new compounds that may be therapeutically beneficial if applied early during disease presentation or in acute disease phases, or as a prophylaxis for subsequent illness or infection.

Targeted metabolomic analysis confirmed NAD(P)HX accumulation in the three patient fibroblast lines. Based on the patient versus control fold changes, the accumulation was much more pronounced in the two cases with ‘Combined NAXD’ deficiency and a neurological presentation (Case 1 and Case 9) than in the cardiac case (Case 5), where we identified compound heterozygosity with one ‘Mito NAXD’ allele and one ‘Combined NAXD’ allele. The more moderate damage accumulation in Case 5 can readily be explained by residual Cyto NAXD activity maintained in the p.(Ala20Phefs*9) variant, which would still permit expression of Mito NAXD. While the pronounced NADHX accumulation in fibroblasts from Case 9 is consistent with the great protein instability that we observed for the corresponding missense allele [p.(Ser235Phe)] and the truncating frameshift mutation on the other allele, it was more surprising to find such a strong effect in the fibroblasts from Case 1, given the high residual enzyme activity observed with the corresponding missense variant [p.(Arg250His)]. In the same fibroblast lines, quantitative proteomics demonstrated a significant decrease in NAXD protein levels, with the degree of NAXD deficiency being most severe in the in utero case (Case 9) followed by the cardiac case (Case 5) and finally the typical PEBEL2 case (Case 1). The proteomic profile of the cardiac and in utero cases was compared against five previously reported PEBEL2 cases that had neurological presentations [12]. The published neurological cases predominantly had increased gluconeogenesis, with decreased mitochondrial TCA and pyruvate metabolism, and mitochondrial translation. In comparison, we identified unique proteomic ‘signatures’ in both the cardiac and in utero cases. Interestingly, the cardiac case had enrichment for DNA repair and cell cycle, and decreased lipid metabolism, which has been reported for another NAXD deficiency case [7] and decreased innate immune system pathways, as identified in a zebrafish NAXD model [38]. However, the in utero case had increased metabolism, and decreased extracellular matrix organisation. This may warrant future research using more accurate disease modelling systems to study the cardiac and neurological presentations of PEBEL2 due to the limitations of fibroblast proteomics studies.

Our work assesses the functional impact of missense variants in *NAXD* that were not previously characterised. The missense variants p.(Ile149Ser), p.(Ser235Phe), and p.(Leu294Pro) led to protein instability or protein insolubility, and the purified protein amounts that could be obtained were insufficient for enzyme activity measurements. More stable protein expression could be achieved with the previously reported missense variants p.(Pro103Leu) [1, 13] and the newly identified p.(Arg250His) variant. Although both variants showed substantial residual S‐NADHX dehydratase activity, they displayed highly reduced thermostability compared to the wild‐type protein, which had been reported for other missense variants before [1]. This provides a plausible association of NAXD protein instability with a fever‐induced clinical onset.

A novel variant in the MTS at p.(Met1?) was identified in three patients (Cases 6, 7 and 8) where Met1 is replaced with leucine. We confirmed that a slightly smaller NAXD protein, likely initiated at Met3, was retained in the cytoplasm but mitochondrial expression was prevented, consistent with previous studies suggesting partial translational leakage from Met1 to Met3 [4]. The association of MTS truncating variants with a cardiac/muscular phenotype is increasing [1, 6, 7, 8]. We hypothesise that muscle tissue, in particular the heart, is especially sensitive to mitochondrial NAD(P)HX damage. It is well established that mitochondrial failure is broadly associated with cardiac manifestations [39], and we see mitochondrial defects in PEBEL2 patients. However, we lack a deep understanding of the underlying pathomechanisms leading to the cardiac and muscular phenotype seen in PEBEL2 individuals. Stress (illness, fever or infection) is believed to be the primary trigger for neurodegeneration in PEBEL2, and emerging evidence associates illnesses and infections with the cardiac and muscular NAXD presentation as well. Therefore, it is critical to determine how stress triggers the key pathological processes underlying disease initiation and progression, eventually leading to the cardiac and muscular decline in PEBEL2.

## 5 Conclusion

In conclusion, improving our understanding of the molecular disease mechanisms in PEBEL2 and the impact of variants on protein function will increase the diagnostic rate for this ultra‐rare condition. Further adding to the complexity is the expanding clinical phenotype of PEBEL2, where we now have more supportive evidence of a cardiac involvement in nine patients, generally associated with variants in the MTS. Hence, there is significant value in *NAXD* being considered for inclusion in other gene panels (e.g., cardiac, fetal anomalies, adult‐onset epilepsy) to facilitate more rapid diagnosis and potentially preventing a fatal disease outcome.

Future work should focus on modelling these distinct clinical sub‐types, for instance by using induced pluripotent stem cells (iPSCs) and differentiating them to disease‐associated cell types. Indeed, iPSC models of NAXD [40] and NAXE [41] are becoming available. Additionally, whole‐organism models such as zebrafish [38] or rodents [42] will allow more comprehensive studies of these disorders, providing unique insights into the pathological effects on the most vulnerable tissues such as the brain and the heart. These iPSC and animal models could also be used to study how external stimuli (e.g., stress or inflammatory triggers) may accelerate disease development. More broadly, these studies could deepen our understanding of other related mitochondrial and metabolic disorders.

## Author Contributions

**Najmesadat Seyedkatouli:** methodology, validation, formal analysis, investigation, writing – original draft, writing – review and editing, visualization. **Liana N. Semcesen:** methodology, validation, formal analysis, investigation, writing – original draft, writing – review and editing, visualisation. **Lucia Gallucci:** methodology, validation, formal analysis, investigation, writing – review and editing, visualisation. **Tim Sikora:** formal analysis, investigation, writing – review and editing. **Jean‐François Conrotte:** formal analysis, investigation, writing – review and editing. **Mei R. M. Du:** methodology, validation, formal analysis, investigation, writing – review and editing, visualisation. **Marat Kasakin:** formal analysis, investigation, writing – review and editing. **Gezime Seferi:** formal analysis, investigation, writing – review and editing. **Licia Corona:** formal analysis, investigation, writing – review and editing. **Martin Jakubec:** formal analysis, investigation, writing – review and editing. **Brunda Nijagal:** formal analysis, investigation, writing – review and editing. **Sajel Lala:** resources, writing – review and editing. **Rebecca D. Ganetzky:** resources, writing – review and editing. **Ana Maria Rodriguez Barreto:** resources, writing – review and editing. **Marina Szlago:** resources, writing – review and editing. **Melanie Wong:** resources, writing – review and editing. **Margit Shah:** resources, writing – review and editing. **James Nurse:** resources, writing – review and editing. **Nicola Foulds:** resources, writing – review and editing. **Shankar Sadagopan:** resources, writing – review and editing. **Ha Nguyen Thu:** resources, writing – review and editing. **Dung Vu Chi:** resources, writing – review and editing. **Khanh Nguyen Ngoc:** resources, writing – review and editing. **Michelle G. de Silva:** resources, writing – review and editing. **Mirana Ramialison:** resources, writing – review and editing. **Fernando Rossello:** resources, writing – review and editing. **MitoMDT Diagnostic Network for Genomics and Omics:** resources, writing – review and editing. **David R. Thorburn:** resources, writing – review and editing. **Matthew Lynch:** resources, writing – review and editing. **Pauline McGrath:** resources, writing – review and editing. **David A. Stroud:** formal analysis, investigation, writing – review and editing. **John Christodoulou:** conceptualization, writing – original draft, writing – review and editing, supervision, project administration, funding acquisition. **Carole L. Linster:** conceptualization, writing – original draft, writing – review and editing, supervision, project administration, funding acquisition. **Nicole J. Van Bergen:** conceptualization, methodology, validation, formal analysis, investigation, resources, data curation, writing – original draft, writing – review and editing, visualisation, supervision, project administration, funding acquisition.

## Funding

The research conducted at the Murdoch Children's Research Institute was supported by the Victorian Government's Operational Infrastructure Support Program. The work was supported by funding from the Mito Foundation to NVB and CLL (G202), an equipment grant to DAS (G189) and a PhD Top‐up scholarship (S021 LNS), the Victorian Health and Medical Research Workforce Project, The Victorian Government, The Victorian Department of Jobs, Precincts and Regions, AAMRI and VESKI Victorian Near‐miss Award Pilot to NVB. The Chair in Genomic Medicine awarded to JC is generously supported by The Royal Children's Hospital Foundation. This research was supported by an Australian National Health and Medical Research Council Investigator Fellowship (GNT2009732 to DAS), a Principal Research Fellowship (GNT1155244 DRT) as well as the Australian Genomics NHMRC Targeted Call for Research grant GNT1113531 and the Australian Medical Research Future Fund (MRFF); Genomics Health Futures Mission (2007959 DRT, 2016030 DAS). The MitoMDT Diagnostic Network for Genomics and Omics acknowledges financial support from the Australian Government's Medical Research Future Fund (MRFF grant reference number MRF2007959), the Mito Foundation and Australian Genomics. Analysis was supported by the Centre for Population Genomics (Garvan Institute of Medical Research and Murdoch Children's Research Institute) and was funded in part by a National Health and Medical Research Council (NHMRC) investigator grant (2009982), the Medical Research Future Fund (MRFF) Genomics Health Futures Mission (2008820). The contents of this published material are solely the responsibility of the authors and do not reflect the views of the Commonwealth of Australia or the NHMRC. The research performed at the Luxembourg Centre for Systems Biomedicine was also supported by the Luxembourg National Research Fund (Fonds National de la Recherche ‐ FNR) through the CORE grant C22/BM/17198760/NAXDivo and the INTER European Joint Programme on Rare Diseases (EJP RD) grant INTER/EJPRD22/17557557/GENOMIT to CLL, and a PhD fellowship to NK within the doctoral training unit ACTIVE (PRIDE19/14063202) under the supervision of C.L.L.

## Conflicts of Interest

The authors declare no conflicts of interest.

## Supporting information


**Figure S1:** Pedigree of the family of Case 6, 7 and 8 and confirmatory Sanger sequencing.
**Figure S2:** Normal brain MRI of Case 6.
**Figure S3:** Fetal MRI and posthumous MRI from Case 9.
**Figure S4:** Enzymatic assay of NAXD missense variants.
**Figure S5:** Relative complex abundance plots for Cases 1, 5 and 9.
**Figure S6:** Bioinformatic pathway analysis of patient fibroblast proteomic data.
**Figure S7:** Pathway comparison of paediatric controls versus NAXD cases.
**Figure S8:** p.Met1? immunofluorescent representative images and Western blots.
**Figure S9:** Full size Western blots.
**Table S1:** Mass transitions and compound dependent source parameters (SCIEX 7500 TQ system).
**Table S3:** Variant classification according to ACMG criteria.
**Table S4:** Kinetic properties of stably expressed NAXD missense variants.
**Table S5:** Summary of quantitative proteomics.
**Table S6:** Primers for site‐directed mutagenesis of missense variants.

**Table S2:** Full clinical summary of all previously reported NAXD and NAXE cases.

## Data Availability

The data that support the findings of this study are available from the corresponding author, upon reasonable request.

## Appendix A Consortia: MitoMDT Diagnostic Network for Genomics and Omics

Shanti Balasubramaniam, Megan Ball, John Christodoulou, David Coman, Alison Compton, Ryan Davis, Lucas Dejong, Yoni Elbaum, Aleksandra Filipovska, Luke Formosa, Roula Ghaoui, Madeleine Harris, Daniella Hock, Nicole Lake, Daniel MacArthur, Pauline McGrath, Elle Martin, Sean Murray, Rocio Rius, Mike Ryan, Amanda Samarasinghe, Liana Semcesen, Stefan Siira, Tegan Stait, Diana Stojanovski, David Stroud, David Thorburn, Aimee Woods.
