# Supplementary material for: NAXD Deficiency: Heterogeneous Phenotypes and Positive Response to Niacin Treatment
Source: J Inherit Metab Dis. 2026 Jul 12;49(4):e70217. doi: 10.1002/jimd.70217 (PMC13358410; doi:10.1002/jimd.70217)
Supplement: Supplementary file 1 — Figure S1: Pedigree of the family of Case 6, 7 and 8 and confirmatory Sanger sequencing. Figure S2: Normal brain MRI of Case 6. Figure S3: Fetal MRI and posthumous MRI from Case 9. Figure S4: Enzymatic assay of NAXD missense variants. Figure S5: Relative complex abundance plots for Cases 1, 5 and 9. Figure S6: Bioinformatic pathway analysis of patient fibroblast proteomic data. Figure S7: Pathway comparison of paediatric controls versus NAXD cases. Figure S8: p.Met1? immunofluorescent representative images and Western blots. Figure S9: Full size Western blots. Table S1: Mass transitions and compound dependent source parameters (SCIEX 7500 TQ system). Table S3: Variant classification according to ACMG criteria. Table S4: Kinetic properties of stably expressed NAXD missense variants. Table S5: Summary of quantitative proteomics. Table S6: Primers for site‐directed mutagenesis of missense variants. [file JIMD-49-0-s002.docx]

A)


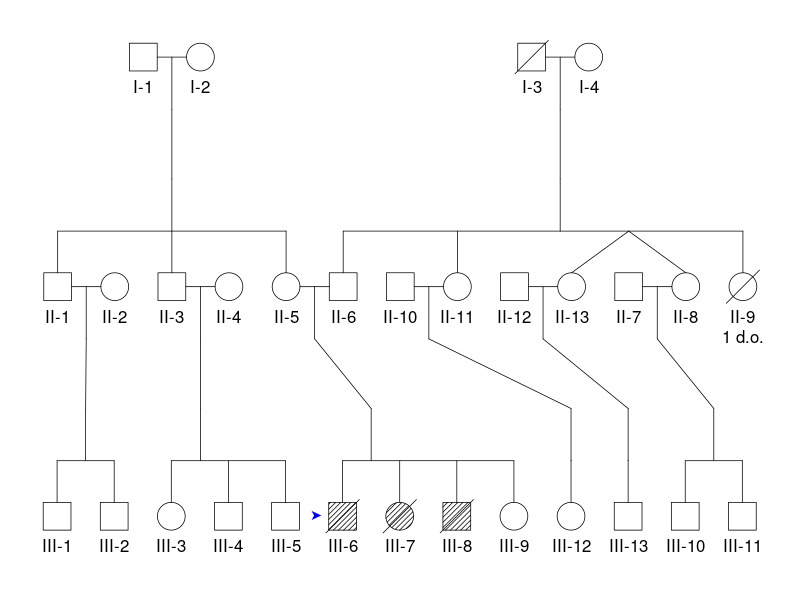


B)

| Proband (III-6) | 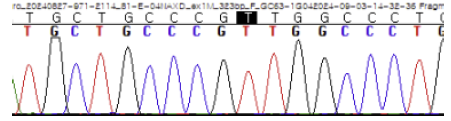 |
| --- | --- |
| Mother (II-6) | 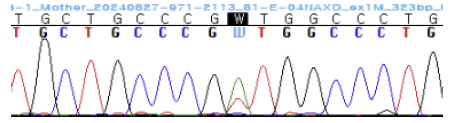 |
| Father (II-5) | 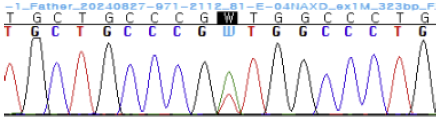 |

**Supplementary Figure 1: Pedigree of the family of Case 6, 7 and 8 and confirmatory Sanger sequencing**

A) Pedigree of the family with the p.Met1? variant. Family member I-3 had sudden death at 49 years old, cause unknown. Proband, Case 6 (III-6) was diagnosed with myocarditis. Case 7 (III-7) died at 4 years old, presumptive diagnosis of myocarditis. Case 8 (III-8) died at 5 years old, presumptive diagnosis of myocarditis. B) Confirmatory Sanger sequencing of c.1A>T; p.Met1? variant.


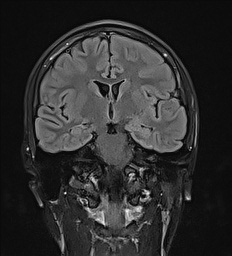


**Supplementary Figure 2: Normal brain MRI of Case 6**


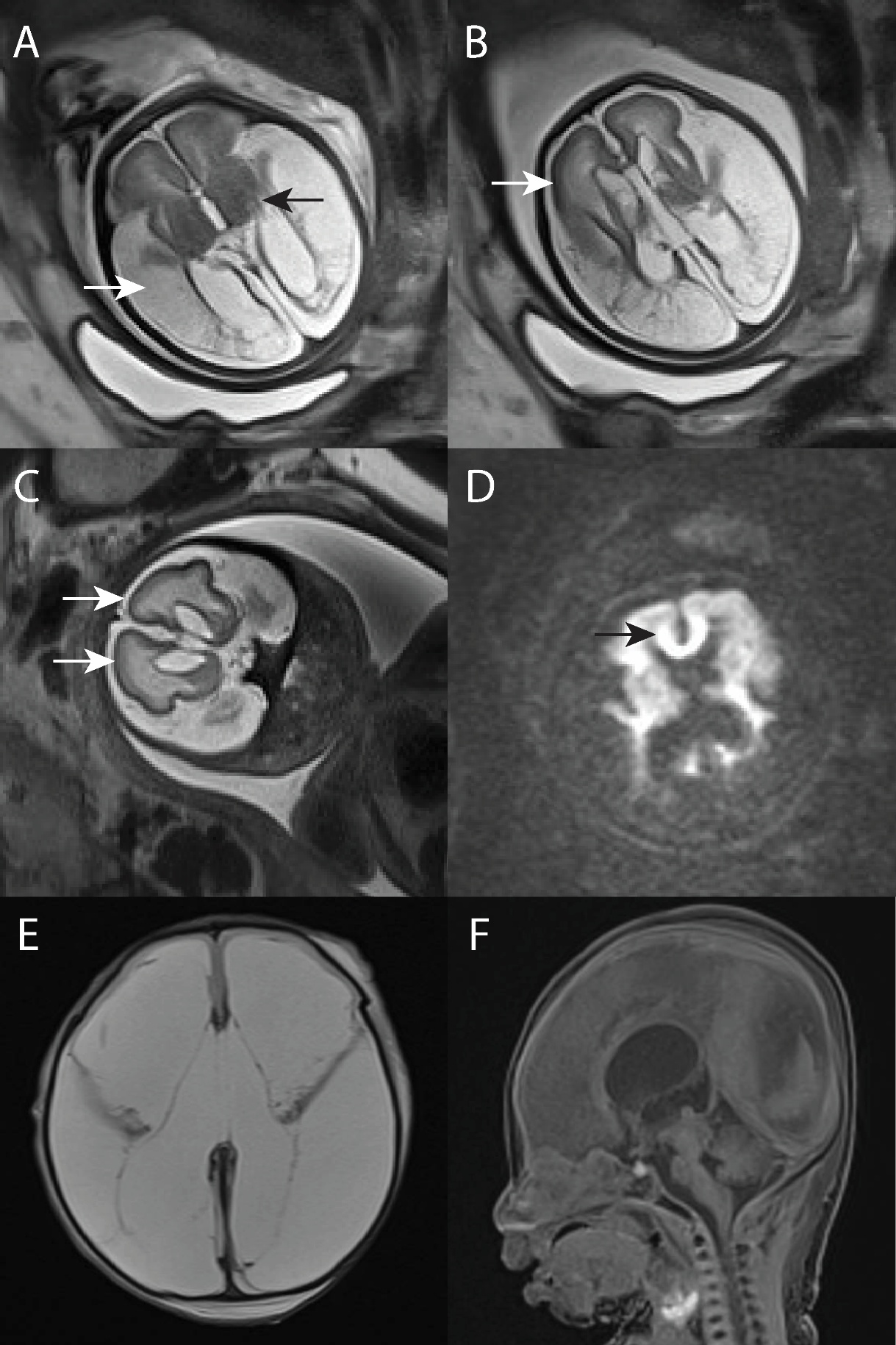


**Supplementary Fig. 3 Fetal MRI and posthumous MRI from Case 9.**

Fetal MRI at 29 weeks was very abnormal and featured extensive cystic encephalomalacia. **A:** Symmetric severe cystic encephalomalacia (white arrow), basal ganglia with abnormal dark appearance (black arrow) on T2 and **B:** ssymmetric bilateral T2 hyperintense white matter (white arrow) at 29 weeks. **C:** Bright frontal white matter on T2 (white arrows) and **D:** diffusion restriction corpus callosum at 29 weeks (black arrow). **E** and **F:** Postmortem MRI at 33 weeks demonstrated extensive gliosis in the ganglia and thalami and a markedly attenuated corpus callosum.

**
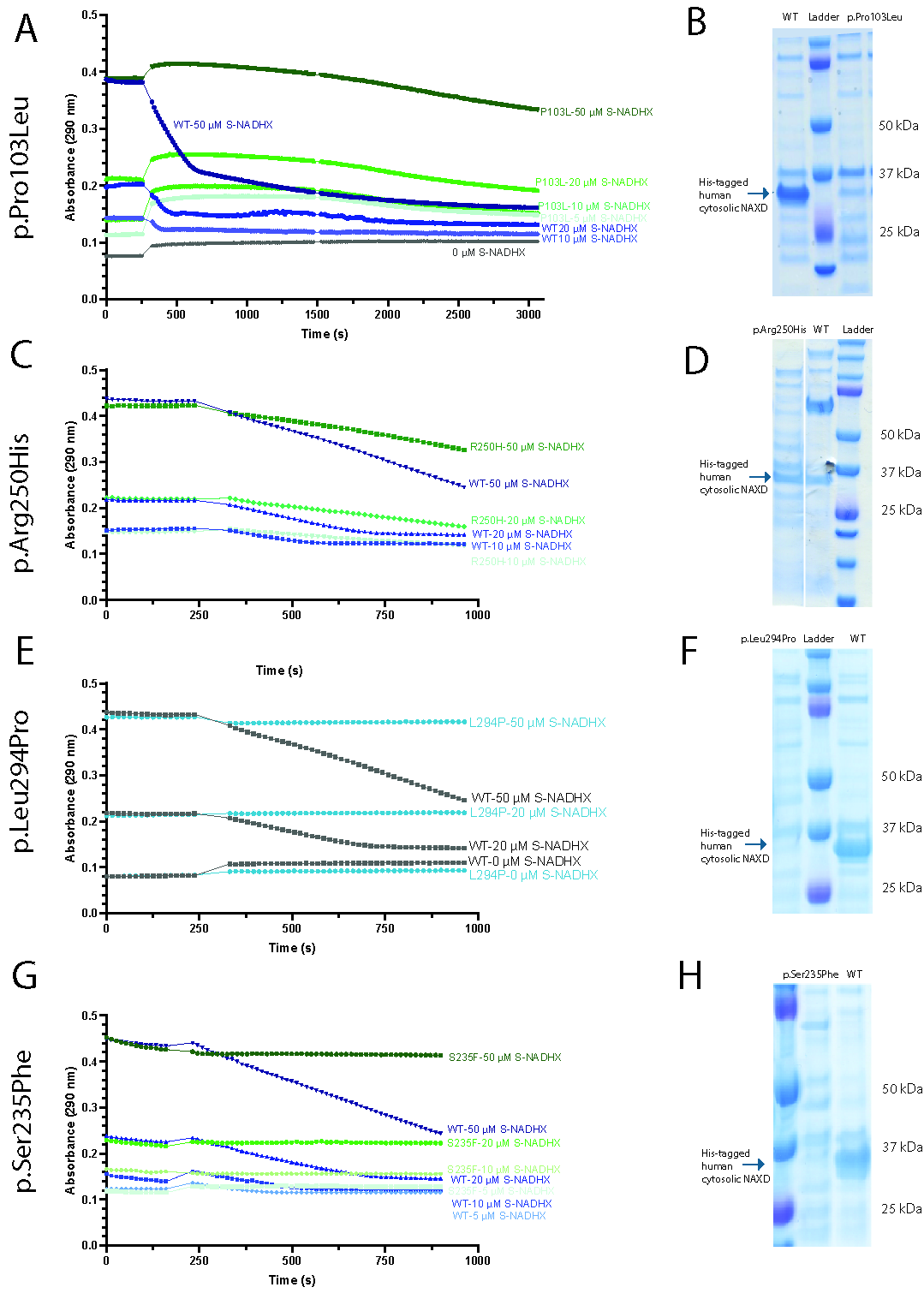
**

**Supplementary Figure 4: Enzymatic assay of NAXD missense variants**

A, C, E, G) Spectrophotometric traces at A_290_, monitoring S-NADHX conversion over time for indicated NAXD proteins incubated with various S-NADHX concentrations. (B, D, F, H): SDS-PAGE analysis of desalted purified human Cyto NAXD proteins.

**
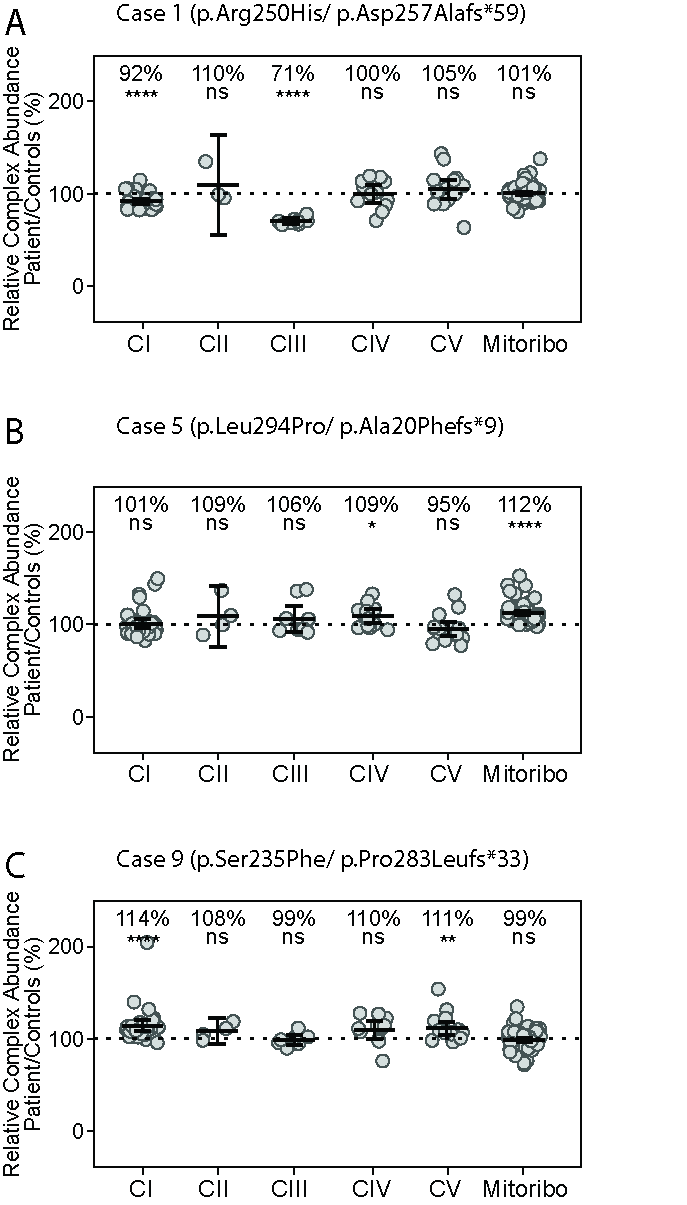
**

**Supplementary Figure 5: Relative Complex Abundance plots for Cases 1, 5 and 9.**

The relative complex abundance (RCA) profiles from quantitative proteomics revealed a significant reduction in mitochondrial OXPHOS complex subunits (CI and CV) and mitoribosomal proteins in fibroblasts from 5 previously reported NAXD cases against controls (n = 5), and a significant reduction in CI and CIII in Case 1. However, there was no decrease in RCA for Case 5 or Case 9. Overall mean values of subunits within each complex were normalised to mitochondrial content levels. # Significant values for all 5 previous *NAXD* cases reported in (Van Bergen et al., 2023) and Supplementary Table 4 are indicated in red and all had a *P* > 0.05. **** *P*<0.0001.

**
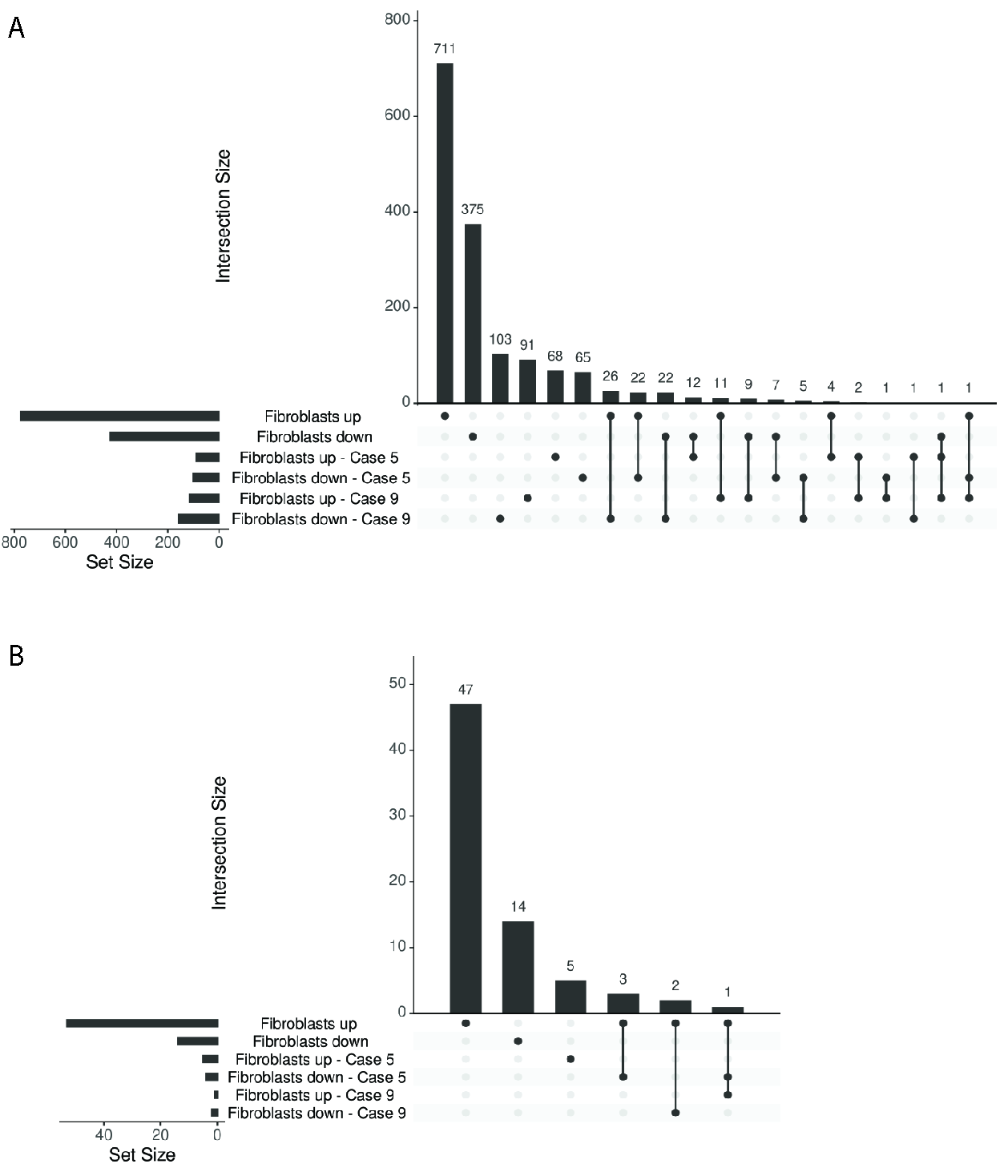
**

**Supplementary Figure 6: Bioinformatic pathway analysis of patient fibroblast proteomic data**

Qualitative analysis of proteomics signatures in five paediatric NAXD fibroblasts (classical PEBEL2 presentation; marked as “Fibroblasts”) from (Van Bergen et al., 2023) versus Case 5 (cardiac) or Case 9 (in utero demise). This demonstrates that most changes are unique to each group, and there is very little cross-talk between datasets. There is almost no overlap between all 3 datasets for either A) differential abundance of individual proteins or B) common pathways based on over-representation analysis.

**
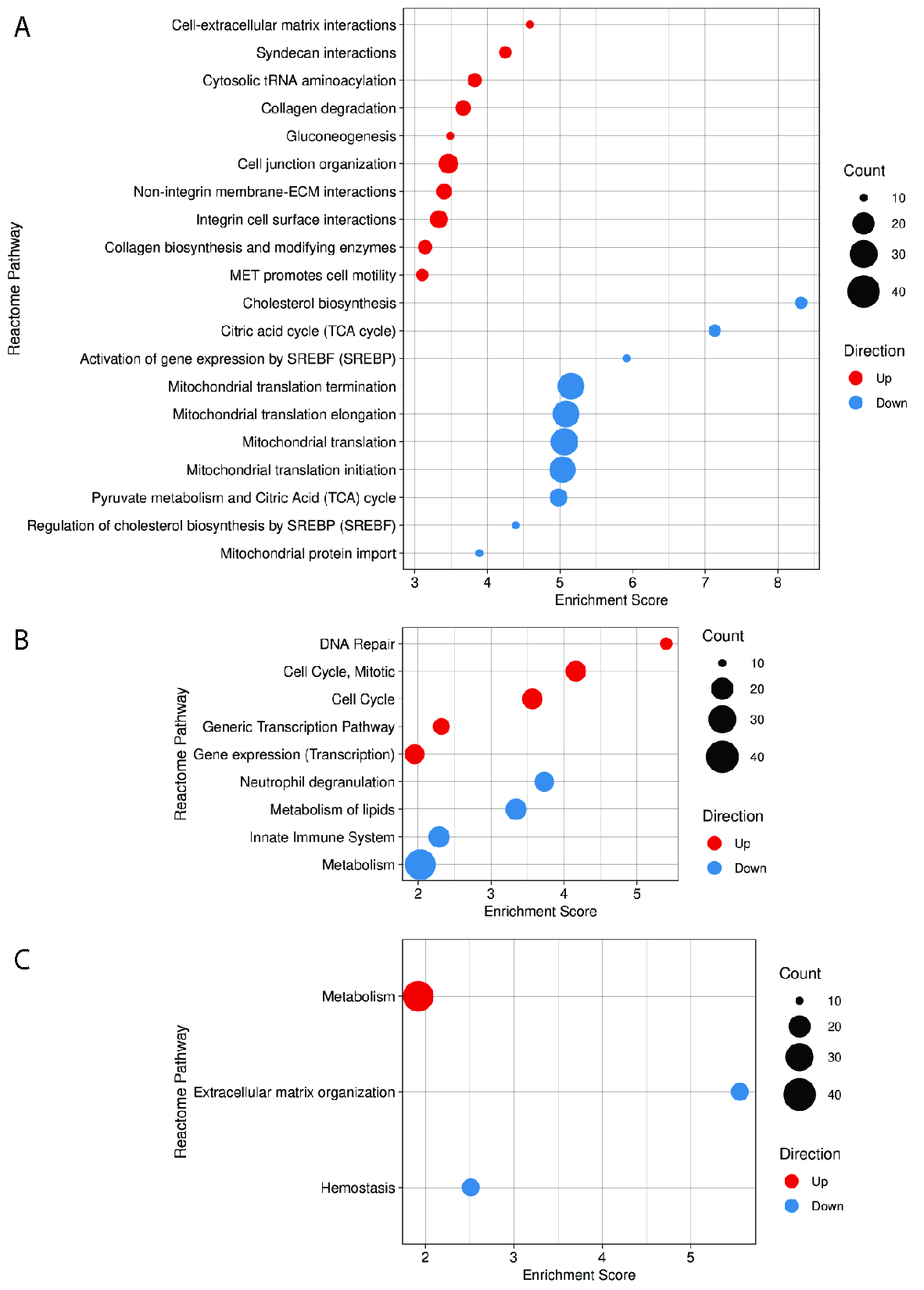
**

**Supplementary Figure 7: Pathway comparison of paediatric controls vs NAXD cases**

Reactome pathways over-represented in sets of up- and down-regulated proteomics signatures in A) five paediatric NAXD fibroblasts (classical PEBEL2 presentation) from (Van Bergen et al., 2023) versus paediatric controls. For this comparison, the top ten pathways for each set of differentially expressed proteins are shown. B) Case 5 (cardiac) vs paediatric controls or C) Case 9 (*in utero* demise) versus paediatric controls. Dot size (Count) indicates the number of input proteins associated with the given pathway. Direction of differential expression is shown by colour. Enrichment Score is the ratio of Count to the number of proteins among all detected background proteins.

**
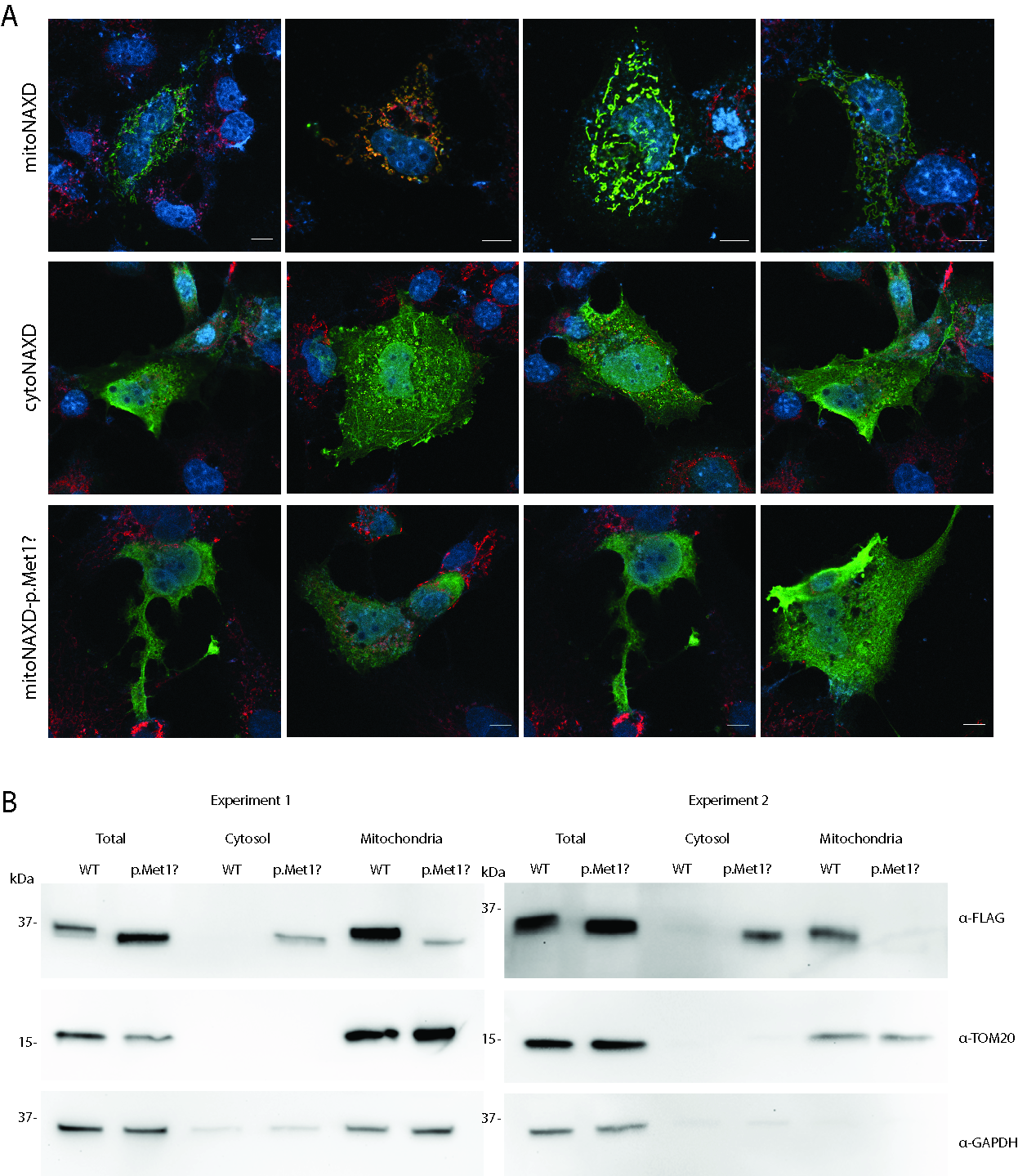
**

**Supplementary Figure 8: p.Met1? immunofluorescent representative images and Western blots**

A) Representative images of COS7 cells that were transfected with mitoNAXD-FLAG, cytoNAXD-FLAG or mitoNAXD p.Met1?-FLAG (p.Met1?) variant introduced by site-directed mutagenesis. Cells were counter-stained for mitochondria (MitoTracker™ Red CMXRos) and nucleus (DAPI), demonstrating cytosolic localisation of p.Met1? NAXD. Scale bar = 10 µm. B) HEK293T cells were transfected and sub-fractionated into cytosolic and mitochondrial fractions. The mitoNAXD protein was present in the mitochondrial fraction, whilst the mitoNAXD p.Met1? protein was expressed mainly in the cytoplasm. GAPDH was used as a marker protein for the cytosol, whilst TOM20 was used as a marker protein for the mitochondria. Two representative experiments are shown.

**
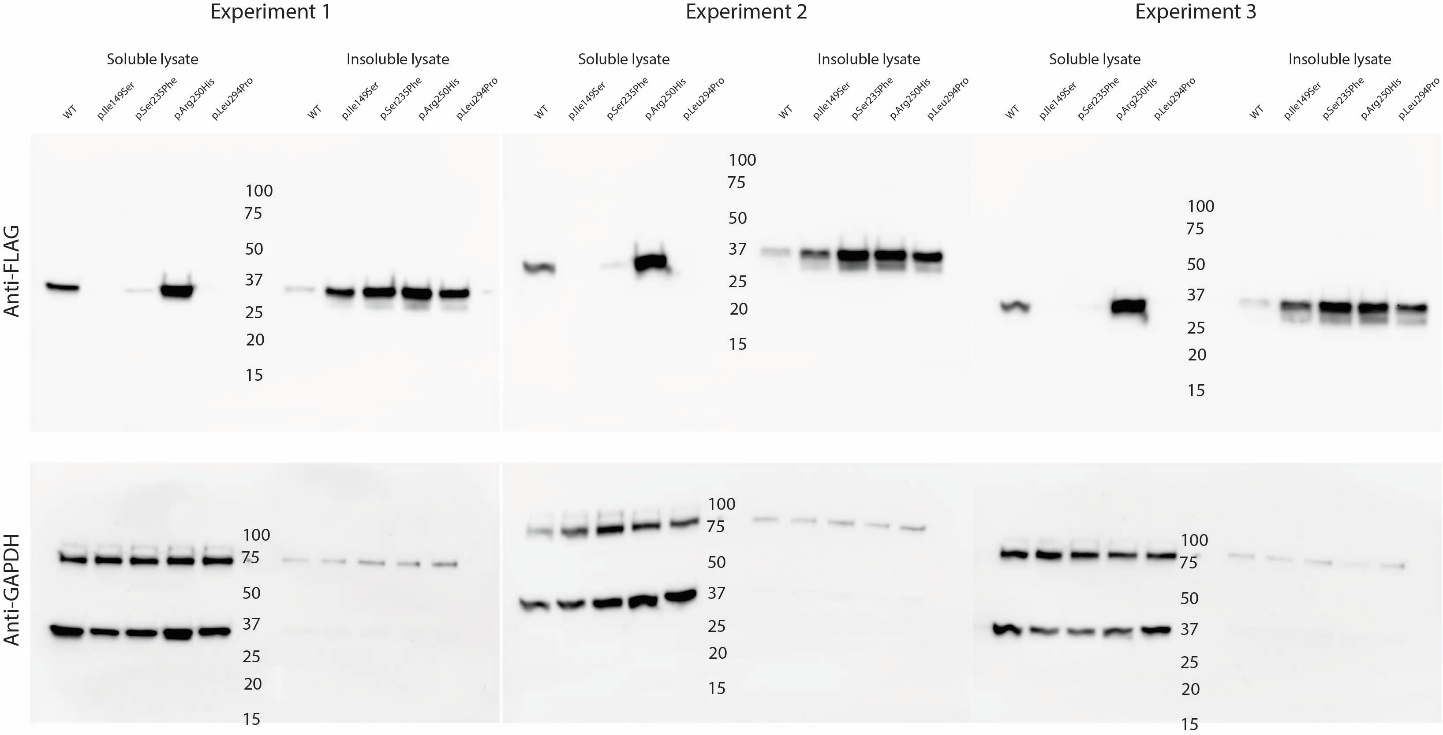
**

**
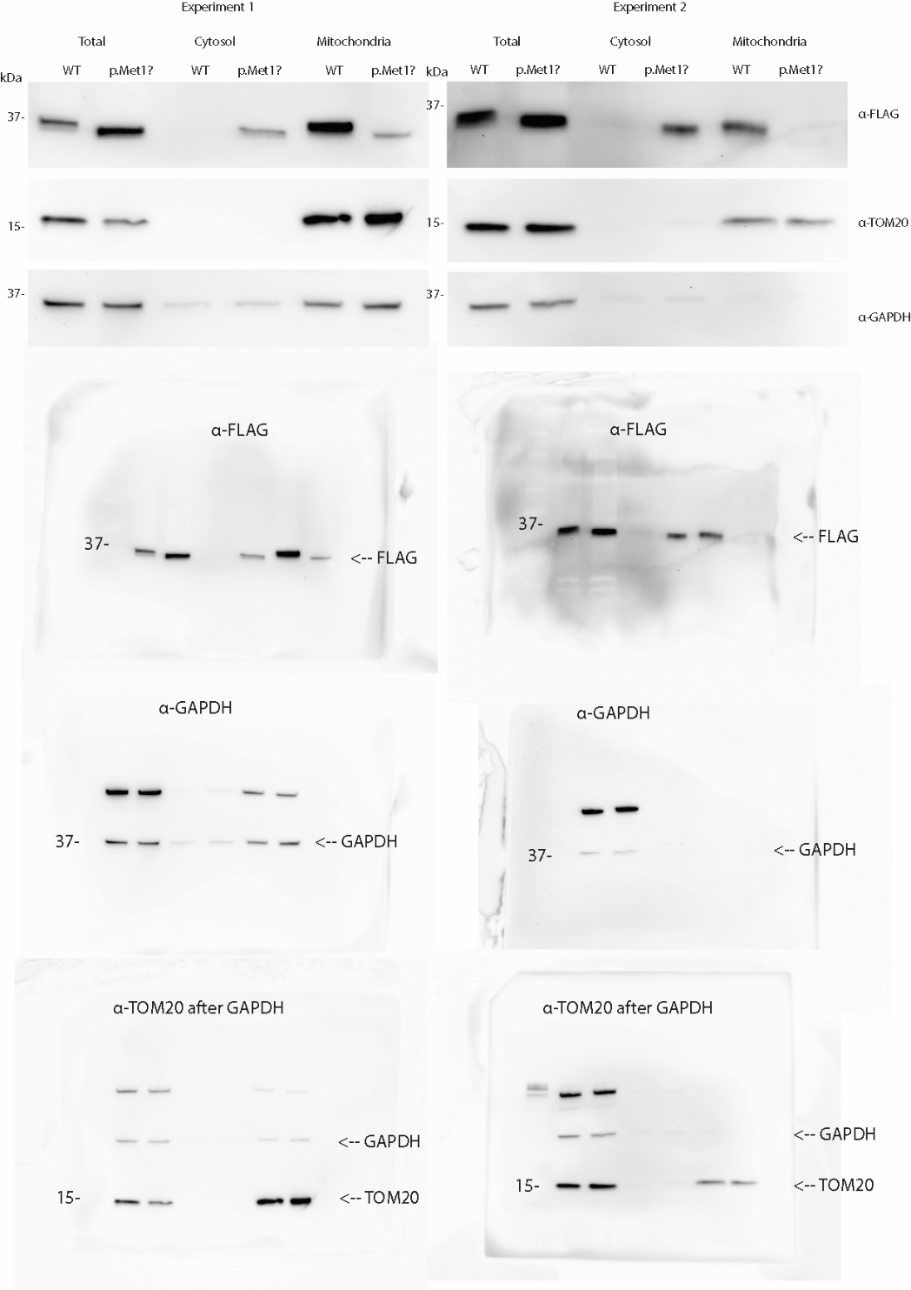
**

**Supplementary Figure 9: Full size Western blots**

Full size Western blots from data presented in Figs 3, 5 and Supplementary Figure 8.

**Supplementary Table 1: Mass transitions and compound dependent source parameters (SCIEX 7500 TQ system).**

| **Group ID** | **Compound ID** | **R_t_**  **(min)** | **Q1**  **(*m*/*z*, Da)** | **Q3**  ***(m*/*z*, Da)** | **DT (ms)** | **EP (V)** | **CE (V)** | **CXP (V)** |
| --- | --- | --- | --- | --- | --- | --- | --- | --- |
| NAD^+^ | NAD^+^_M1 | 4.55 | 664.05 | 428.08 | 25 | 10 | 37 | 9 |
| NAD^+^ | NAD^+^_M2 | 4.55 | 664.05 | 542.06 | 25 | 10 | 23 | 11 |
| NADH | NADH_M1 | 5.94 | 666.13 | 649.1 | 25 | 10 | 23 | 15 |
| NADH | NADH_M2 | 5.94 | 666.13 | 514.07 | 25 | 10 | 35 | 14 |
| R-NADHX | R-NADHX_M1 | 4.62 | 666.09 | 649.16 | 25 | 10 | 24 | 16 |
| R-NADHX | R-NADHX_M2 | 4.62 | 666.09 | 514.03 | 25 | 10 | 35 | 37 |
| S-NADHX | S-NADHX_M1 | 4.06 | 666.06 | 649.15 | 25 | 10 | 24 | 17 |
| S-NADHX | S-NADHX_M2 | 4.06 | 666.06 | 514.07 | 25 | 10 | 34 | 12 |
| c-NADHX | cNADHX_M1 | 7.82 | 666.1 | 649.09 | 25 | 10 | 26 | 18 |
| c-NADHX | cNADHX_M2 | 7.82 | 666.1 | 514.04 | 25 | 10 | 37 | 15 |
| thio_NAD | thio_NAD_M1 | 9.44 | 680.12 | 428.07 | 25 | 10 | 37 | 31 |
| thio_NAD | thio_NAD_M2 | 9.44 | 680.12 | 136.02 | 25 | 10 | 54 | 22 |

**Supplemental Table 2: Full clinical information for NAXD and NAXE cases**

See separate file due to size of table.

**Supplementary Table 3: Variant classification according to ACMG criteria**

| **cDNA position  (NM_001242882.1)** | **P notation  (NP_001229811)** | **Inheritance** | **Final review of pathogenicity (ACMG)** | **Evidence*** | **Variant type** |
| --- | --- | --- | --- | --- | --- |
| c.749G>A | p.(Arg250His) | Parental | Pathogenic (II) | PM3, PP4, PM2, PM5, PP3, PS3, PM1, PP2, PP1-S | Missense |
| c.770del | p.(Asp257Alafs*59) | Parental | Pathogenic (Ia) | PM2, PVS1, PS3, PM1, PP1, PM3, PP4 | Frameshift |
| c.922C>T | p.(Arg308Cys) | Parental | Pathogenic (II) | PM2, PM5, PP3, PS3, PP1-S, PM3, PP4 | Missense |
| c.794_798dup | p.(Val267Serfs*51) | Parental | Pathogenic (Ia) | PM2, PVS1, PM4, PP3, PP1-S, PM1, PM3, PP4 | Frameshift |
| c.446T>C | p.(Ile149Ser) | Parental | Likely pathogenic (IV) | PM2, PP3, PM1, PP1, PM3, PP4 | Missense |
| c.881T>C | p.(Leu294Pro) | Parental | Pathogenic (IIIa) | PM2, PP3, PS3, PM1, PP2, PM3, PP4 | Missense |
| c.51_54delAGAA | p.(Ala20Phefs*9) | Parental | Pathogenic (Ia) | PM2, PVS1, PS1, PM4, PP3, PS3, PP1-M, PM3, PP5, PP4 | Frameshift with loss of MTS; expression of cNAXD at Met3 |
| c.1A>T | p.Met1? | Parental | Pathogenic (Ia) | PM2, PVS1, PS1, PM4, PP3, PS3, PP1-S, PM3, PP4 | Start loss; expression of cNAXD at Met3 |
| c.704C>T | p.(Ser235Phe) | Parental | Pathogenic (II) | PM2, PM5, PP3, PS3, PP2, PS2, PM3, PP4 | Missense |
| c.848del | p.(Pro283Leufs*33) | Parental | Pathogenic (Ia) | PM2, PVS1, PM4, PP3, PS3, PM1, PM3, PP4 | Frameshift |

**Supplementary Table 4: Kinetic properties of stably expressed *NAXD* missense variants**

|  | Vmax  (µmol/min/mg protein) | | Km  (µM) | |
| --- | --- | --- | --- | --- |
|  | Average | SD | Average | SD |
| Wild-type | 0.90 | 0.21 | 15.10 | 2.71 |
| p.(Arg250His) from this study | 0.76 | 0.31 | 14.28 | 6.78 |
| p.(Pro103Leu) from Van Bergen et al 201 | 0.40* | 0.31 | 9.31 | 3.35 |

Data are means ± SD, n ≥ 3 per measurement from at least three independent experiments. NADHX dehydratase activity was assayed spectrophotometrically by monitoring the consumption of S-NADHX. Statistical significance was determined using one-way ANOVA without correction for multiple comparisons, significance is *P<0.05.

**Supplementary Table 5: Summary of quantitative proteomics**

The relative complex abundance (RCA) profiles from quantitative proteomics revealed a significant reduction in mitochondrial OXPHOS complex subunits (CI and CV) and mitoribosomal proteins in fibroblasts from 5 previously reported NAXD cases against controls (n = 5), and a significant reduction in CI and CIII in Case 1. However, there was no decrease in RCA for Case 5 or Case 9 (also see Supplementary Fig. 5). Overall mean values of subunits within each complex were normalised to mitochondrial content levels. The range of the mean ratio of each complex for the 5 previously reported cases is shown as a percentage value. # Significant values for all 5 previous cases reported in (Van Bergen et al., 2023) all had a *P* > 0.05. **** *P*<0.0001.

| **Case** | ***NAXD* variants** | **NAXD peptides detected** | **Mitochondrial OXPHOS Respiratory Chain (% of Controls)** | | | | | **Mitoribosome (% of Controls)** |
| --- | --- | --- | --- | --- | --- | --- | --- | --- |
|  |  |  | **CI** | **CII** | **CIII** | **CIV** | **CV** |  |
| Previous 5 NAXD cases | Various. See (Van Bergen et al., 2023) | 1 - 9 | 79 – 93  # | 124 - 164 | 91 - 111 | 67 - 97 | 86 – 91  # | 63 – 92  # |
| Case 1 | p.(Arg250His) and p.(Asp257Alafs*59) | 9 - 12 | 92 **** | 110 | 71 **** | 100 | 105 | 101 |
| Case 5 | p.(Leu294Pro) and p.(Ala20Phefs*9) | 7 - 10 | 101 | 109 | 106 | 109 | 95 | 112  **** |
| Case 9 | p.(Ser235Phe) and p.(Pro283Leufs*33) | 1 - 4 | 114  **** | 108 | 99 | 110 | 111  **** | 99 |
| Controls |  | 9 - 12 | 100 | 100 | 100 | 100 | 100 | 100 |

**Supplementary Table 6: Primers for site-directed mutagenesis of missense variants**

| **Site of mutagenesis** | **Primer sequence (5’ to 3’)** |
| --- | --- |
| cytoNAXD L294P SDM F | CACTGCCTGGTGGGAGAGCAGGCGC |
| cytoNAXD L294P SDM R | GCGCCTGCTCTCCCACCAGGCAGTG |
| cytoNAXD I149S SDM F | CCTTTGACACTTCCAAACTGCCCTGGACATTTCTG |
| cytoNAXD I149S SDM R | CAGAAATGTCCAGGGCAGTTTGGAAGTGTCAAAGG |
| cytoNAXD R268H SDM F | CCCTCCACACCTGTGGCTGCTGCCTTC |
| cytoNAXD R268H SDM R | GAAGGCAGCAGCCACAGGTGTGGAGGG |
| cytoNAXD S235F SDM F | CTGCTGGCCGTTGAAGAGGATGTCGCG |
| cytoNAXD S235F SDM R | CGCGACATCCTCTTCAACGGCCAGCAG |
| cytoNAXD L294P SDM F | CACTGCCTGGTGGGAGAGCAGGCGC |

**Full clinical reports**

***Case 1: Typical PEBEL2 presentation with positive response to niacin***

An 8-month old male patient presented at 6 months of age with neuroregression, noted to have abnormal brain MRI upon admission to ICU with seizures. He had choreoathetotic movements and spasticity, and has always had some motor weakness. No cold symptoms were noted upon ICU submission, but he tested positive for parainfluenza upon admission, and required intubation. His brain MRI noted encephalopathic changes, basal ganglia changes, a stroke-like lesion, which was transient and did "resolve"/migrate on repeat imaging a few weeks later. A few weeks after ICU submission, he started to develop a rash in his mouth, on his face and around his groin. During the course of investigations, WGS analysis identified compound heterozygous variants in *NAXD.*

Pharmacologic doses of Niacin (B3) treatment (10mg/kg/day) were commenced via nasogastric tube based on previous studies (Manor et al., 2022; Zhou et al., 2019) (he was 8 months of age) in addition to Biotin 10mg/kg, Thiamin 40mg/kg intravenously,

CoQ10 - 15 mg/kg/day and carnitine - 100 mg/kg/day. Arginine had been given due to concerns with MELAS from MRI findings. The improvement after starting niacin was very drastic, he had already required CPR several times and was decompensating very fast, after niacin, he stabilized rapidly. The rash improved and subsequently, his clinical status also to improve. He was able to breathe on room air, and was more responsive to stimuli, but continued receiving nasogastric feeds. He does have residual spasticity/neuroirritability. His clinical course has been complicated by micro hemorrhages on repeat MRI thought to be due to a transient pancytopenia that he developed during his stay, which is thought to be the reason for his neuro symptoms.

Currently he is 2 years and 3 months of age and is on the following cocktail;

- N-acetylcysteine 50 mg daily --> 100 mg if next set of liver/kidney labs look good

- Thiamine 100 mg daily

- Biotin 10 mg daily

- CoQ10 150 mg daily

- Riboflavin 50 mg daily

- Niacin 50 mg daily --> 100 mg if next set of liver/kidney labs look good

- Niacinamide 50 mg daily

He is still on ASDs but has been able to wean off a few of them and seems to be more alert and interactive and less irritable. He’s still G tube dependent but as per last visit was eating one meal by mouth completely daily, and is able to suck from a bottle. He’s still not speaking but babbling and making sounds. He is almost completely independently rolling. He will sit with support. He can bear weight on his legs when placed in a standing position and can hold/reach for toys. He is not having overt seizures, and the mother notes that he may be having some absence seizures a few times a week. He continues to have tremors/dystonic movements. He continues to regain milestones and is now 27 months of age.

***Case 2: Typical PEBEL2 presentation untreated, with demise***

This previously healthy 15-month boy was first admitted to ICU with a 22-day history of febrile illness, gingivostomatitis, diarrhea and feeding difficulties. At admission he was consciousness impaired, and presented with oral mucositis and skin ulcers, mainly in perianal and periureteral areas. Severe pancytopenia due to bone marrow involvement was prominent. Human Herpesvirus 6 (HVS6)-positive PCR in cerebrospinal fluid (CSF) and blood supported the diagnosis of invasive HVS6 illness. His condition partially improved after treatment with ganciclovir, amphotericin, and fluconazole and he was discharged at day 40 of admission.

At 20 months, the patient experienced a febrile illness and developed neurological worsening, loss of milestones, progressive ataxia, abnormal limb movements, and ocular apraxia-opsoclonus. Additional studies were conducted, (MRI, EEG, blood, urine and CSF) with no diagnosis confirmation. A possible "immune-mediated encephalitis” was suspected and after gamma globulin treatment, partial and temporary improvement was observed.

Subsequently, there was a worsening of the febrile condition. Opsoclonus, dystonic-choreoathetoid movements, and severe mucositis were more evident. An extensive interdisciplinary evaluation was conducted involving oncology, immunology, infectious diseases, hematology, ophthalmology, and toxicology.

A mito-cocktail with folinic acid, biotin, CoQ10, carnitine, riboflavin, and thiamine were empirically prescribed. Despite these efforts, the patient continued to experience progression of both neurological and systemic symptoms.

Further investigations revealed sideroblasts in the bone marrow, retinitis pigmentosa in the fundus oculi, and bilateral symmetric encephalopathy with involvement of both white and grey matter in the MRI. Additionally, there was a notable restriction of diffusion and an increase in lactic acid in spectroscopy.

He died at the age of 23 months with a probable diagnosis of "Uncharacterized Mitochondrial Disease" but without resolution of a genetic cause. Retrospective genetic investigations after his sibling was diagnosed (Case 3) reported the same two variants in *NAXD* gene as Case 3 (detailed below).

***Case 3: Typical PEBEL2 presentation with positive response to niacin***

A 2-years-and-9-months boy was admitted to our clinic for study due to acute ataxia. He is the third child of healthy, non-consanguineous parents and the younger sibling of Case 2. He was born via caesarean section due to maternal hypertension at 37 weeks, weighing 2,425 grams. Neurodevelopmental progress was appropriate for his age. He had a history of herpangina at 3 months of age and two episodes of outpatient-managed bronchospasms at 17 months.

The condition that prompted the hospitalization began a few days before with frequent falls that worsened in the last 24 hours, presenting with progressive difficulty in walking and making gait impossible. During hospitalization, he developed intention tremors, choreoathetotic movements and irritability, and it was impossible for him to sit without assistance, nor to walk. He was reported to have a COVID-19 infection 20 days before admission. Initial studies, including laboratory tests such as complete blood count, liver and kidney function, serum electrolytes, blood glucose, CPK, lactate, ammonia, and urine toxicology screening, were normal. The cerebrospinal fluid study, including virological analysis, cell count, proteins, glucose, and lactate, also yielded normal results. Neuroimaging with CT and MRI of the central nervous system with spectroscopy, and contrast-enhanced spinal MRI showed no pathological findings. Additional evaluations, including abdominal and renal ultrasound, fundoscopy, and haematological assessment, revealed no abnormalities. Metabolic investigations, including amino acids, organic acids, and acylcarnitines, were also negative.

Given the family history of a deceased sibling (Case 2) with a similar onset, without a confirmed etiology, empirical treatment with thiamine, biotin, riboflavin, carnitine, folic acid, and CoQ10 was initiated. The patient was discharged with partial clinical improvement.

At that time a genetic panel was performed, including genes related to leukodystrophy and leukoencephalopathy, revealing two compound heterozygous variants in *NAXD*.

Based on the genetic findings and consultation with experts on NAXD disorders, niacin therapy (initially 200mg (100mg twice a day) then increased to 300mg/day) was initiated at 8 months of age. In this case, it showed a beneficial therapeutic effect in resolving neurological symptoms without harmful side effects. The patient is progressing with marked improvement in motor skills.

After the initiation of niacin treatment, the patient experienced febrile infectious processes on several occasions without showing a decline in general condition or neurological deterioration. There was no need for further hospitalizations. Currently, at 6 years of age, he shows good height and weight progress. Motor development is appropriate, but there is a lag in the language area and hyperactivity.

***Case 4: Early PEBEL2 presentation with positive response to niacin***

Case 4 was a previously well child of consanguineous parentage with no relevant family history. She presented at the age of 12 months acutely unwell with febrile illness, severe diarrhoea and encephalopathy associated with pancytopenia (with neutrophil count 0) and raised systemic and CSF inflammatory markers. Abnormal vacuolation of erythroid and neutrophil precursors were seen on the bone marrow biopsy. She appeared to respond to steroids, but not GM-CSF, broad spectrum antimicrobials or IVIG. She was discharged well after a month but represented two months later with profound vomiting, diarrhoea, increasing lethargy and floppiness, but without encephalopathic. She was again pancytopenia and responded to steroid. Sirolimus and prophylactic cotrimoxazole were commenced whilst awaiting the results of genetic testing for a potential immune dysregulation associated disorder. Two months later, at 17 months of age, she again presented with severe diarrhoea, vomiting and fever after minor viral respiratory symptoms. She again responded to steroids, but in retrospect, her mother felt that early commencement of parenteral nutrition was the intervention correlating best with the clinical improvement during each preceding episode. Genetic testing results returned soon after admission, identifying a homozygous missense variant in *NAXD* (NM_00124882.2:c.446T>G, p.(Ile149Ser)) which was predicted to be probably damaging by *in silico* analysis (Polyphen score of 0.969 out of 1). She was commenced on niacin treatment (40mg bd) immediately after genetic diagnosis, and there was a dramatic improvement in her symptoms, the trajectory of which was much faster than previously experienced with steroids, and without commencing parenteral nutrition. Niacin was well tolerated except for occasional flushing immediately after the dose. At last follow-up 6 months later, Case 4 was in good health, developmentally normal, with no further metabolic or immune dysregulation associated deteriorations despite several mild viral respiratory tract infections which had previously precipitated a flare.

***Case 5: Cardiac NAXD presentation with positive response to niacin***

A previously well 4 year 11-month-old girl was admitted to their local hospital with a chest pain, increased work of breathing and a 4-day history of cough and cold symptoms. She had deranged liver function tests, with an elevated creatinine kinase (CK) and troponin levels. The initial diagnosis was viral myocarditis but her cardiac function rapidly deteriorated over the next 72 hours leading to severe LV dysfunction. She developed cardiogenic shock and was intubated and ventilated and transferred to a regional paediatric intensive care unit. Her lactate was 8 mmol/Land she was in a peri-arrest state requiring three ionotropes. Her liver function tests, CK and troponin levels were elevated and she was placed on extracorporeal membrane oxygenation (ECMO) in view of severity of cardiac status. She remained on ECMO for 13 days before discontinuation with spontaneous improvement in cardiac function. She developed ischaemia of the small bowel with perforation, leading to laparotomy, resection and end ileostomy. She was started on an ACE inhibitor and beta blocker and continued to improve; she was discharged approximately two months after her initial presentation. Investigations for metabolic causes of cardiomyopathy were normal; an initial respiratory panel showed a low level of adenovirus but was negative on repeat. A cardiomyopathy gene panel was reported as normal.

Four months later she presented to her local hospital with a 1-day history of leg pain, chest pain, sore throat and lethargy, she tested positive for COVID-19. She was promptly transferred to a regional cardiology centre for further management, and echocardiogram showed mildly depressed left ventricle systolic function, visually estimated ejection fraction (EF) ~ 40-50% and biplane EF 45%. She deteriorated over the next 72 hours with vomiting, hypotension, tachycardia and a worsening lactic acidosis. Repeat echocardiogram showed worsening left ventricle function and she was commenced on veno-arterial ECMO and this continued for 11 days. Cardiac function improved over this time, and she continued to make a slow recovery.

After her second ECMO, a brain MRI was undertaken and compared to an MRI approximately 4 weeks earlier. There was no evidence of recent infarct. Old right MCA territory infarct. There was generalised widening of the extra-axial spaces and proportional distension of the lateral ventricles, and several micro-haemorrhages in supra and infratentorial white matter consistent with ECMO history.

Rapid whole genome testing was undertaken in view of the second presenting episode and two compound heterozygous variants in the *NAXD* gene were identified. She was commenced on Niacin (Niacin as Nicotinamide 15mg/kg/day in 2 doses) in view of the suspicion of NAXD deficiency and a bespoke emergency plan developed with a low carbohydrate, high fat energy mix.

At follow-up at 6.5 years, she is on an ACE inhibitor, beta blocker and twice daily Niacin. She reports episodic dizziness that has been disruptive and may be related to her Niacin although this has improved with a change in ACE inhibitor preparation. She is not back in full time school and missed a whole year of education, but her parents feel she is beginning to catch up. Her physical assessment was unremarkable with no evidence of myopathy. She has had 3 febrile illnesses since commencing Niacin, including one admission to hospital with vomiting, her CK and lactate were normal during the admission, and her latest echocardiogram shows that overall left ventricle systolic function appears good. EF by 2D Teich = 73%. Visual EF = 65 - 70%. She continues with a stoma due to concerns over the risk of major abdominal surgery and there is no body of evidence to inform this decision***.***

***Case 6, 7 and 8: Family with Cardiac presentation***

A 14-year-old male patient (Case 6) was admitted to the hospital due to substernal and epigastric chest pain accompanied by mild exertional dyspnea. Just before admission, the patient had been examined at a private hospital where elevated liver enzymes, muscle enzymes, and Troponin I were noted, but no treatment was given. He is the first child of a non-consanguineous family, born full term with a birth weight of 3.4 kg, and had normal physical and psychomotor development. In 2022, he had been diagnosed with acute rhabdomyolysis following a febrile illness, with markedly elevated muscle and liver enzymes (CK 27,271 U/L and GOT/GPT 1127/529 U/L), which resolved with treatment. Since then, he had occasionally experienced chest pain and exertional dyspnea, although electrocardiogram (ECG) and echocardiography had shown no abnormalities. The family history was notable for sudden unexplained deaths: the paternal grandfather died at 49 years of age from unknown cause, and both younger siblings died, presumably due to myocarditis (Supplementary Fig. 2).

At admission, the patient was alert, afebrile, with stable respiration and circulation. Initial physical examination revealed no focal neurological signs, no muscle weakness, and no muscle hypertrophy. Cardiovascular and respiratory examination showed no abnormalities, with pain localized only to the substernal region. Initial laboratory findings showed CK 6324 U/L, CK-MB 227 U/L, Troponin I 0.079 ng/mL, lactate 4.9 mmol/L, NH₃ 62.1 µmol/L, GOT/GPT 367/154 U/L; blood gas analysis showed no acidosis, with normal electrolytes and calcium. Echocardiography demonstrated normal chamber dimensions, good contractility, and no pericardial effusion; however, the ECG showed T-wave abnormalities in leads V3–V6. Previous brain MRI had been normal (Supplementary Fig. 3). With elevated muscle and cardiac enzymes and elevated blood lactate, in the context of prior rhabdomyolysis, the initial differential diagnoses included acute myocarditis, primary myopathy, and mitochondrial metabolic disorders. Myocarditis was the leading diagnosis to be excluded, given the chest pain, elevated cardiac enzymes, and ECG changes, although echocardiography did not show contractility impairment or effusion. Nevertheless, an underlying metabolic myopathy or mitochondrial disorder remained highly suspected. The patient was treated with continuous intravenous glucose infusion (glucose infusion rate 2–4 mg/kg/min), combined with L-carnitine, arginine, B vitamins, and coenzyme Q10. During treatment, CK decreased from 6324 U/L to 3119 U/L and then 2567 U/L; Troponin I increased from 0.079 to 0.512 ng/mL and then decreased to 0.324 ng/mL; lactate fluctuated slightly between 4.9 and 5.04 mmol/L. Clinically, the patient remained alert, with stable respiration and hemodynamics for the first six days, without signs of heart failure or infection. However, on the morning of day 7, he suddenly collapsed in the bathroom, became comatose, and suffered respiratory and cardiac arrest. Despite nearly an hour of unsuccessful cardiopulmonary resuscitation, the patient was declared dead.

On retrospective review of the medical records of his younger brother — who had died a year earlier with a presumptive diagnosis of myocarditis — the onset was noted to be acute with fatigue, vomiting, and pallor. At admission, the child was alert but tachycardic, with gallop rhythm and mild dyspnea. Echocardiography revealed severely reduced ejection fraction of 27% and dilated chambers. Initial laboratory results showed elevated Troponin I (0.825 ng/mL), elevated lactate (5 mmol/L), and elevated proBNP (1500 pg/mL); these parameters continued to rise rapidly in subsequent days, along with increased liver enzymes, metabolic acidosis, and coagulopathy. Despite intensive treatment, the patient did not survive. Metabolic disorder screening by tandem mass spectrometry (MS/MS) was within normal limits. Trio-WES (whole-exome sequencing of the parents and the patient) and a mitochondrial gene panel performed at that time did not detect any clearly pathogenic variants.

Case 7 was a younger sibling of Case 6. He was 5 years of age when he died with cardiac manifestations, diagnosed as myocarditis. The onset was acute, with fatigue, vomiting and pallor. Upon admission, the child was tachycardic, with a gallop rhythm and mild dyspnoea. Echocardiography revealed a severely reduced ejection fraction of 27% and dilated chambers. Despite intensive treatment, the patient did not survive. He was homozygous for the same NAXD variant that was identified as in Case 6.

Case 8 was a younger sibling of Cases 6 and 7. She died at the age of 4 years with a diagnosis of myocarditis. Limited clinical information was available for this case, and genetic testing was not possible. A fourth younger sibling of Cases 6, 7 and 8 is not a carrier of the variant and is clinically well and is homozygous for the wild-type NAXD sequence.

With the complete clinical presentation of the three siblings from the same family, we reanalyzed the family’s genetic data and identified a homozygous variant c.1A>T (p.Met1?) in the *NAXD* gene, classified as pathogenic. This variant is associated with NAD(P)HX repair deficiency – an extremely rare metabolic disorder that can cause damage to the myocardium, skeletal muscle, and central nervous system following triggering factors such as fever or metabolic stress.

***Case 9: In utero demise***

This baby was the first pregnancy to a healthy consanguineous couple of Pakistani descent. They were third cousins, and there was no family history. The couple underwent extended carrier screening (Mackenzie Mission) in 2020 and were low risk. The pregnancy was conceived naturally. The first trimester was unremarkable. At the routine morphology ultrasound at 20 weeks gestation, isolated mild bilateral ventriculomegaly was identified. Routine bedside ultrasound at 28 weeks identified an abnormal appearance of the cerebral cortex, with extensive supratentorial abnormalities, confirmed on fetal MRI at 29 weeks. Due to the family’s beliefs, amniocentesis testing was not performed. No illnesses were noted during the pregnancy.

Fetal MRI performed at 28 week 5 days demonstrated microcephaly (brain BPD < 3rd centile) mild ventriculomegaly (right lateral ventricle 11.7 mm, left lateral ventricle 10.3 mm), and extensive symmetric cystic encephalomalacia of the occipital, temporal and parietal lobes. The white matter of the frontal lobes was abnormal, with T2 hyperintense signal. There was diffusion restriction of the corpus callosum and the residual occipital lobe, suggesting an active destructive process (Supplementary Fig. 1A – D). Posterior fossa structures were normal.

The mother went into spontaneous preterm labour at 33 weeks, and a male baby was delivered still born. No notable cutaneous or neurological features were noted at birth. An autopsy was not performed at the family request.

A postmortem brain MRI was performed on day 1 after birth. This demonstrated complete cystic encephalomalacia of all supratentorial structures (cortex, white matter and deep grey nuclei) with sparing of the posterior fossa (cortex, white matter and deep grey nuclei, Supplementary Fig. 1E, F). A trio whole genome was performed which identified compound heterozygous *NAXD* variants.

**NAXD variant classification**

***NAXD reference variant***

NAXD variants here are reported according to the most abundant transcript NM_001242882.1, which generates both Mito NAXD and Cyto NAXD (Van Bergen et al., 2019).

***Case 1*** had compound heterozygous variants in *NAXD*; a maternally inherited missense variant (NM_001242882.1: c.749G>A: p.(Arg250His)) and a paternally inherited frameshift variant (NM_001242882.1: c.770del: p.(Asp257Alafs*59)). The p.(Arg250His) missense variant is in a highly conserved region of the NAXD protein, and is 5 amino acids away from a critical NAD(P)HX binding site. In silico analysis (Polyphen2) predicted this variant to be probably damaging, with a score of 1 out of 1, and is 5 amino acids away from a critical NAD(P)HX binding site however this variant was initially reported as a variant of uncertain significance (VUS). The paternally inherited p.(Asp257Alafs*59) frameshift variant is predicted to result in an abnormal protein length, loss of a putative NAD(P)HX and ATP binding site (Figure 1) and was initially reported to be likely pathogenic. In addition, other clinically significant *NAXD* variants are reported in this region (Van Bergen et al., 2019) and this study.

***Case 2 and 3*** are siblings and both had compound heterozygous variants in *NAXD*; a missense variant (NM_001242882.1: c.922C>T: p.(Arg308Cys)) and a frameshift variant (NM_001242882.1:c.794_798dup: p.(Val267Serfs*51). The missense variant p.(Arg308Cys) causes enzyme kinetic impairments and thermostability (Van Bergen et al., 2019) and is classed as pathogenic. The variant p.(Val267Serfs*51) creates a premature stop signal, and significant frameshift resulting in loss of a critical ATP binding site (Figure 1). This variant is not present in population databases and has not been reported associated with disease. This variant has been reported in ClinVar in a patient with mild neurosensory hearing impairment, progressive encephalopathy, chorea, seizures and pancytopenia and was recorded as likely pathogenic. This variant resides in a region of *NAXD* where other clinically significant *NAXD* variants including p.(Ala317Leufs*64) (Van Bergen et al., 2019) and those in this report.

**Case 4** had a homozygous missense variant in *NAXD* (NM_001242882.1; c.446T>C, p.(Ile149Ser)) and was maternally and paternally inherited. This variant has not been reported in ClinVar, and is absent in population database gnomAD in homozygous state and only 3 heterozygous states. The variant was initially classified as a VUS. In silico analysis (Polyphen2) predicted this variant to be probably damaging, with a score of 0.969 out of 1.

***Case 5*** had compound heterozygous variants in *NAXD;* a maternally inherited frameshift variant (NM_001242882.2: c.54_57del: p.(Ala20Phefs*9)) and a paternally inherited missense variant (NM_001242882.2: c.881T>C: p.(Leu294Pro)). The p.(Ala20Phefs*9) has been previously reported in both a homozygous state (Van Bergen et al., 2019) and as one of two compound heterozygous variants (Borna et al., 2020), has been reported several times in ClinVar associated with PEBEL2, and is likely pathogenic. The p.(Ala20Phefs*9) variant results in loss of Mito NAXD expression, but retention of Cyto NAXD (Borna et al., 2020), suggesting utilisation of Met3 (Figure 1). The missense variant, p.(Leu294Pro) is two amino acids away from a highly conserved ATP binding site, so could likely affect enzyme kinetics, and is classified as probably damaging by *in silico* analysis (Polyphen score of 0.999 out of 1), has not been reported in ClinVar previously and is absent in population database gnomAD in homozygous or heterozygous states. The variant was initially classified as a VUS.

***Case 6, 7 and 8*** had a homozygous variant in *NAXD* (NM_001242882.1; c.1A>T, p.Met1?) which was predicted to lead to loss of Met1 of the mitochondrial propeptide, but allow translation at Met3, generating cytoplasmic NAXD. This variant has not been reported in ClinVar, and is absent in population database gnomAD in homozygous state and only 3 heterozygous states. The variant was predicted to be pathogenic.

***Case 9:*** had compound heterozygous variants in *NAXD;* a maternally inherited truncating variant (NM_001242882.2: c.848del; p.(Pro283Leufs*33)) and a paternally inherited missense variant (NM_001242882.2: c.704C>T; p.(Ser235Phe)). The p.(Pro283Leufs*33) is predicted to result in a frameshift, generating truncated protein and loss of an ATP binding site. This variant has not been reported in ClinVar, and is absent in population database gnomAD in homozygous or heterozygous states. The missense variant, p.(Ser235Phe) is predicted to be benign by *in silico* analysis, with a Polyphen score of 0.334 out of 1, and is in a highly conserved region, with a major amino acid change. Both of these variants were initially reported as a VUS.

**Supplementary Methods**

**Fibroblast culture**

Primary cultures of fibroblasts from cases and age-matched paediatric controls maintained in a medium containing high-glucose DMEM (Gibco, 11960-044), 1X Glutamax (Gibco, 35050-061), 10% FBS (Gibco, 10500-064), 100 units/mL penicillin, and 100 µg/mL streptomycin (Gibco, 15140-122) in Flasks (Thermo Fisher Scientific, Nunc EasYFlask Nunclon Delta Surface) at 37°C with 5% CO2. The cells were passaged, with a medium change, once or twice a week when they reached confluency. Due to poor cell viability and growth, the FBS concentration was increased to 15% for Case 9 fibroblasts and 10 ng/mL Basic FGF was added for expanding the cells. The cultivation conditions were readjusted to the ones for all other cell cultures upon seeding the Case 9 fibroblasts for metabolite extraction and analysis.

**Putative NAD(P)HX metabolite analysis in fibroblasts (Case 1)**

Chemicals obtained included: Acetonitrile Hypergrade for LCMS LiChrosolv, 2.5L (Merck, Australia). Water was Milli Q grade. Formic Acid, 99.0+%, Optima™ LC/MS Grade (Thermo, Australia).

Adherent cells were harvested by aspirating the media and washing the cells with 37°C PBS. The PBS was aspirated off the cells, and the plate was placed on a dry ice-ethanol bath to quench the cells.

Polar metabolite extraction was performed by adding 300µl of cold extraction solvent 9:1 Methanol/Water (containing 2µM 13C-sorbitol, 2µM 13C,15N-Valine, 2µM 13C,15N-AMP, 2µM 13C,15N-UMP), the cells were scraped with a cell lifter and lysed into the extracted solvent. The extraction solvent was transferred into a 1.5ml microcentrifuge tube, vortexed vigorously and incubated for 5mins at 4°C on the thermomixer to ensure lysis of all cell membranes. Finally, the lysate was harvested by centrifuging the cell extract at max speed for 10 mins at 4°C to remove any cell debris. The extracted lysate was transferred into HPLC vials containing glass inserts in preparation for LCMS analysis.

Metabolite separation was performed on Vanquish Horizon UHPLC system (Thermo Scientific) coupled to an Orbitrap IQ-X Tribrid mass spectrometer (Thermo Scientific) for metabolite detection by Metabolomics Australia. The chromatography conditions were modified from Drummond KJ, et al (Drummond et al., 2025). In brief, separation was performed on Merck SeQuant ZIC‐HILIC column (150 mm × 4.6 mm, 5 μm particle size) maintained at 25°C, using a binary gradient consisting of solvent A: 20 mM ammonium carbonate (pH 9.0; Sigma–Aldrich) and solvent B: 100% ACN. The gradient run was as follows: time (t) = 0.0 min, 80% B; t = 0.5 min, 80% B; t = 15.5 min, 50% B; t = 17.5 min, 30% B; t = 18.5 min, 5%; t = 21.0 min, 5% B; t = 23–33 min, 80% at a solvent flow rate of 300 μl/min.

Metabolite detection was performed on a Orbitrap IQ-X Tribrid Mass Spectrometer (Thermo Scientific) coupled to heated electrospray ionisation (H-ESI) source with the following conditions: sheath gas flow 40 arbitrary units (Arb), auxiliary gas flow 10 Arb, sweep gas flow 1 Arb, ion transfer tube temperature 275 °C, and vaporizer temperature 320 °C. The RF lens value was 35%. Data was acquired in positive polarity with spray voltages of 3500 V.

Data analysis was performed on TraceFinder Software (Thermo Scientific™) and El-Maven (https://www.elucidata.io/el-maven). Level 1 metabolite identification, according to the Metabolite Standard Initiative (Fiehn et al., 2007), was based on matching accurate mass and retention time to the 550 authentic standards in the Metabolomics Australia (MA) in‐house library. Note, chemically pure standards of S-NADHX and R-NADHX were not available in the reference laboratory.

**NAD(P)HX metabolite analysis in fibroblasts (Case 5)**

**Cell culture**

Fibroblasts were seeded in basal media containing high-glucose DMEM with 1X Glutamax, 10% dialyzed FBS (Gibco, 26400044), 100 units/mL penicillin, and 100 µg/mL streptomycin, at a density of 1.25×10^5^ cells/well into standard 6-well plates (Thermo Fisher Scientific, Nunclon Delta Surface, 140685). The cells were cultivated for 120 hours, by which time cells were approximately 90% confluent. Case 9 line seeding density was increased to 1.6×10^5^ cells/well, to account for the slower growth rate. Viable cell counting was performed in an automated cell counter (Thermo Fisher Scientific, Invitrogen, Countess 3), using Trypan Blue stain 0.4% (Invitrogen, T10282) according to the manufacturer’s instructions.

**Metabolite extraction**

For metabolite extraction, culture media was aspirated and cells were gently washed with pre-warmed (37°C) Dulbecco’s phosphate buffered saline (DPBS, Gibco, 14200-075). The extraction fluid consisted of Methanol:20mM Tris buffer pH 8 = 4:1 (Methanol, Carl Roth, AE71.2; Tris-base, Thermo Fisher Scientific, BP138603) containing 1 μg/ml of thionicotinamide adenine dinucleotide (thio-NAD) as internal standard (Sigma-Aldrich, Merck, T7375) and it was bubbled with nitrogen (N₂) to minimize oxidation of labile metabolites and pre-cooled (-20°C). After addition of 500 µL of extraction fluid, the cells were immediately placed on a cooling plate and incubated for 1 minute. Cells were then collected via scraping and transferred to a 2 ml tube prefilled with 200 µL of Chloroform at room temperature (Carl Roth, 7331.2) and incubated for 10 min at 15°C and 2000 rpm (Eppendorf ThermoMixer Comfort). Upon mixing, additional 200 µL of Chloroform and 260 µL Tris buffer pH 8 precooled (4°C) were added. The mixture was vortexed thoroughly (10 seconds), then centrifuged for 5 minutes at 4°C and 21,000 g to achieve phase separation. A defined volume of 600 µL of the upper polar phase was transferred to a new 2 mL tube prefilled with 1 mL of MilliQ water (room temperature) and snap frozen in liquid nitrogen prior to lyophilisation (FreeZone 2.5 Plus, Labconco) overnight. The samples were either used directly for metabolite analysis or stored at -80°C until analysis.

Protein pellets (derived from the interphase after phase separation) were dried overnight at 4°C using a refrigerated centrifugal vacuum concentrator (CentriVap 7310000, Labconco) and used for protein M1ification. Protein pellets were resuspended in 40 µL of 1% SDS and incubated for 10 minutes at 95°C and 2000 rpm (Eppendorf ThermoMixer Comfort). Total protein amounts were M1ified with the Pierce BCA Protein Assay Kit (Thermo Fischer Scientific, 23227) according to the manufacturer’s instructions.

**Targeted NAD(P)X and NAD(P)H measurements by LC-MS/MS**

The LC-MS system consisted of an Exion LC, for metabolite separation, coupled to a 7500 Triple Quad MS (SCIEX) equipped with an Optiflow Pro Ion Source for metabolite detection.

Lyophilized polar extracts were reconstituted in 100 µL of 50 mM ammonium acetate (Ammonium acetate ≥99%, HiPerSolv CHROMANORM® for LC-MS, VWR, 84885.18) in Milli-Q® water (IQ 7000 with Q-POD® and LC-Pak®, meeting ≥18.2 MΩ·cm resistivity and ≤5 ppb TOC for LC-MS suitability), and filtered through Phenex-RC 4 mm Syringe filters into amber LC-MS glass vials with micro-inserts.

Analyte separation was achieved by reversed phase chromatography using a Polaris 3 C18-A column 150 x 3 mm x 3 µm (A2001150X030, Agilent), equipped with a SecurityGuard™ ULTRA Cartridges, UHPLC C18 3.0mm ID Columns (AJ0-8775, Phenomenex), maintained at a temperature of 25 °C. The autosampler was set at 4 °C. Mobile phases comprised 50 mM ammonium acetate in Milli-Q water/ACN (97:3, v/v) as eluent A, and 100% ACN as eluent B, with a flow rate of 300 μl/min. The LC method included a 10-min isocratic delivery of 100% eluent A, followed by a 2-min linear gradient to 99% eluent B; then, a 5-min isocratic delivery of 99% eluent B, and finally, a 8-min re-equilibration phase at the initial 100% eluent A. The total method duration was 25 minutes, with a total scan time of 0.756 seconds. Injection volume was 20 µL.

The ion source gases 1 and 2 were maintained at 30 and 50 psi, respectively. The curtain gas was at a pressure of 40 psi, collision gas at 8, and source temperature held at 350 °C. Spray voltage was set to 2000 V in positive ion mode. Target compounds were measured in scheduled multiple reaction monitoring mode. The target cycle time was 500 ms with a maximum dwell time of 25 ms per transition. Specific parameters for each target analyte are detailed in Supplementary Table 2. Mass spectrometric data were acquired and analysed using SCIEX OS (Version 3.4.0.19154, SCIEX 7500 system).

### Cloning of missense variants for expression in mammalian cells

*NAXD* missense variants were introduced into a pDONR221 plasmid containing the sequence for the cytosolic NAXD isoform via site-directed mutagenesis (Agilent, primers listed in Supplementary Table 6). Donor vectors were cloned into pDEST26-N-FLAG (Addgene, #79549) using the LR Clonase II Kit (ThermoFisher, #11791020) according to the manufacturer’s instructions. Plasmids were isolated from bacterial cultures (QIAGEN, #12145) and sequence confirmed with Plasmidsaurus. 5x10^5 cells were seeded per well of a 6-well plate and transfected with 2µg of plasmid using 4.2µL Lipofectamine™ 2000 for 24 hours. Cells were then harvested in 120ul of RIPA, sonicated, incubated on ice for 20 min, then centrifuged at 4°C for 20 min at 13,000g. The supernatant (“soluble fraction”) was transferred to a new tube and the pellet was resuspended in equal volume of RIPA and sonicated briefly to resolubilize.

Equal volumes of samples were mixed with 2x loading buffer + β-mercaptoethanol, sonicated then heated at 95°C for 5 minutes. Fractions were loaded into a 4 - 15% Mini-PROTEAN® TGX™ Precast Gel (#4561085, Bio-Rad) and run at 150V for ~60 minutes. The gel was wet-transferred onto a methanol-activated PVDF membrane at 100V for 60 minutes before being blocked in 5% skim milk in PBS-T. Primary antibodies to GAPDH (1:5000, #G9545, Merck) and FLAG (1:1000, #F1804, Merck) were incubated at 4°C overnight. Primaries were washed off with PBS-T and membranes were incubated with HRP-conjugated secondary antibodies (anti-mouse-HRP; GEHENA931-1ml, Bio-Strategy, or anti-rabbit-HRP; 7074S, Cell Signaling) before being exposed to Clarity™ Western ECL Substrate and imaged using a ChemiDoc.

### Cloning of p.Met1? variant for expression in mammalian cells

The p.Met1? variant was introduced into the pDONR221 plasmid containing mitochondrial NAXD using Agilent’s Lightning Site Directed Mutagenesis Kit as per the manufacturer’s protocol and custom primers (forward 5'-gaggacccagggccaagaagcctgctttttt-3' and reverse 5’-aaaaaagcaggcttcttggccctgggtcctc-3’). Colonies were screened via Plasmidsaurus, and amplified in LB broth for plasmid isolation using the QIAGEN Plasmid Midi Kit. Variant and wild-type vectors were flipped into a C-terminal FLAG-tagged expression vector (#118372, Addgene) for expression in mammalian cells using Gateway™ LR Clonase™ II Enzyme mix (#11791020, ThermoFisher) according to the manufacturer’s protocol. Colonies were again screened and plasmids were again purified for use in transfection. Full plasmid maps are in Supplementary information.

### Isolation of mitochondria and Western blot of p.Met1? variant

HEK293T cells were cultured in high-glucose DMEM containing 10% fetal bovine serum and 1% penicillin/streptomycin. 2x10^6 cells were seeded in a 10cm² dish and transfected with 12ug of plasmid using Lipofectamine™ 2000 for 24 hours. Mitochondria were isolated according to previous methods with minor modifications (Johnston et al., 2002). Cells were collected in PBS, centrifuged at 800g for 5 minutes then resuspended in ice-cold 1ml solution B (20mM HEPES pH 7.6, 220mM Mannitol, 70mM sucrose, 1mM EDTA, 0.5mM PMSF) then incubated on ice for 15 minutes (total protein fraction). Cells were disrupted with 20 strokes of a homogenizer then centrifuged at 800g for 5 min at 4°C. Supernatant (cytosol fraction) was centrifuged at 10,000 g for 10 minutes, the pellet resuspended in Solution B, centrifuged again then the pellet was resuspended in 50 µl of Solution B (mitochondrial fraction). Equal volumes of samples were mixed with 2x loading buffer + b-mercaptoethanol, sonicated then heated at 95°C for 5 minutes. Fractions were loaded into a 10% Mini-PROTEAN® TGX™ Precast Gel (#4561034, Bio-Rad) and run at 150V for ~60 minutes. The gel was wet-transferred onto a methanol-activated PVDF membrane at 100V for 60 minutes before being blocked in 5% skim milk in PBS-T. Primary antibodies to TOM20 (1:1000, 11802-1-AP ProteinTech), GAPDH (1:5000, #G9545, Merck) and FLAG (1:1000, #F1804, Merck) were incubated at 4°C overnight. Primaries were washed off with PBS-T and membranes were incubated with HRP-conjugated secondary antibodies (anti-mouse-HRP; GEHENA931-1ml, Bio-Strategy, or anti-rabbit-HRP; 7074S, Cell Signaling) before being exposed to Clarity™ Western ECL Substrate and imaged using a ChemiDoc.

### Immunofluorescence of p.Met1? variant

COS7 cells were cultured in high-glucose DMEM containing 10% fetal bovine serum and 1% penicillin/streptomycin. 5x10^4 cells were seeded in each well of a 24-well plate and transfected with 300ng of plasmid using Lipofectamine™ 2000 for 24 hours. The next day, cells were incubated in media containing 50nM MitoTracker™ Red CMXRos for 30 minutes at 37°C before being washed with PBS and fixed in 4% paraformaldehyde for 20 minutes at room temperature. Cells were permeabilized with 0.25% Triton-X for 10 minutes at room temperature and then blocked with 1% BSA in PBS. Cells were incubated with antibodies for 1 hour at room temperature; anti-FLAG (1:1000, #F1804, Merck) primary antibody, and anti-mouse 488 (1:2000, #A-11029, ThermoFisher) secondary antibody. Coverslips were mounted onto microscope slides using ProLong™ Gold Antifade Mountant with DNA Stain DAPI (#P36935, ThermoFisher) and imaged using a LSM900 confocal microscope (ZEISS) and 40x oil immersion objective.

**Expression plasmids**

**pDEST-mitoNAXD-WT-C-FLAG**

**Met1 (mitoNAXD)**

acaagtttgtacaaaaaagcaggcttcatggccctgggtcctcgctgtggggcaatccgg
 T  S  L  Y  K  K  A  G  F  **M**  A  L  G  P  R  C  G  A  I  R 
gcttgcagacgagttttagaaagagcgttttcgctacgtaaagcacattcgataaaggat
 A  C  R  R  V  L  E  R  A  F  S  L  R  K  A  H  S  I  K  D 
atggaaaatactttgcagctggtgagaaatatcatacctcctctgtcttccacaaagcac
**Met3 (cytoNAXD)**

**M**  E  N  T  L  Q  L  V  R  N  I  I  P  P  L  S  S  T  K  H 
aaagggcaagatggaagaataggcgtagttggaggctgtcaggagtacactggagcccca
 K  G  Q  D  G  R  I  G  V  V  G  G  C  Q  E  Y  T  G  A  P 
tattttgcagcaatctcagctctcaaagtgggcgcagacttgtcccacgtgttctgtgcc
 Y  F  A  A  I  S  A  L  K  V  G  A  D  L  S  H  V  F  C  A 
agtgcggccgcacctgtgattaaggcctacagcccggagctgatcgtccacccagttctt
 S  A  A  A  P  V  I  K  A  Y  S  P  E  L  I  V  H  P  V  L 
gacagccccaatgctgttcatgaggtggagaagtggctgccccggctgcatgctcttgtc
 D  S  P  N  A  V  H  E  V  E  K  W  L  P  R  L  H  A  L  V 
gtaggacctggcttgggtagagatgatgcgcttctcagaaatgtccagggcattttggaa
 V  G  P  G  L  G  R  D  D  A  L  L  R  N  V  Q  G  I  L  E 
gtgtcaaaggccagggacatccctgttgtcatcgacgcggatggcctgtggctggtcgct
 V  S  K  A  R  D  I  P  V  V  I  D  A  D  G  L  W  L  V  A 
cagcagccggccctcatccatggctaccggaaggctgtgctcactcccaaccacgtggag
 Q  Q  P  A  L  I  H  G  Y  R  K  A  V  L  T  P  N  H  V  E 
ttcagcagactgtatgacgctgtgctcagaggccctatggacagcgatgacagccatgga
 F  S  R  L  Y  D  A  V  L  R  G  P  M  D  S  D  D  S  H  G 
tctgtgctaagactcagccaagccctgggcaacgtgacggtggtccagaaaggagagcgc
 S  V  L  R  L  S  Q  A  L  G  N  V  T  V  V  Q  K  G  E  R 
gacatcctctccaacggccagcaggtgcttgtgtgcagccaggaaggcagcagccgcagg
 D  I  L  S  N  G  Q  Q  V  L  V  C  S  Q  E  G  S  S  R  R 
tgtggagggcaaggggacctcctgtcgggctccctgggcgtcctggtacactgggcgctc
 C  G  G  Q  G  D  L  L  S  G  S  L  G  V  L  V  H  W  A  L 
cttgctggaccacagaaaacaaatgggtccagccctctcctggtggccgcgtttggcgcc
 L  A  G  P  Q  K  T  N  G  S  S  P  L  L  V  A  A  F  G  A 
tgctctctcaccaggcagtgcaaccaccaagccttccagaagcacggtcgctccaccacc
 C  S  L  T  R  Q  C  N  H  Q  A  F  Q  K  H  G  R  S  T  T 
acctccgacatgatcgccgaggtgggggccgccttcagcaagctctttgaaaccgaccca
 T  S  D  M  I  A  E  V  G  A  A  F  S  K  L  F  E  T  D  P 
gctttcttgtacaaagtggttcgatcttacacgggatccgattacaaggatgacgacgat
 A  F  L  Y  K  V  V  R  S  Y  T  G  S  D  Y  K  D  D  D  D 
aagggcgcagattacaaggatgacgacgataaggcatga
 K  G  A  D  Y  K  D  D  D  D  K  A  -

**pDEST-mitoNAXD-p.Met1?-C-FLAG**

**p.Met1?**

acaagtttgtacaaaaaagcaggcttc**t**tggccctgggtcctcgctgtggggcaatccgg

 T  S  L  Y  K  K  A  G  F  **L**  A  L  G  P  R  C  G  A  I  R 
gcttgcagacgagttttagaaagagcgttttcgctacgtaaagcacattcgataaaggat
 A  C  R  R  V  L  E  R  A  F  S  L  R  K  A  H  S  I  K  D

**Met3 (cytoNAXD)**
atggaaaatactttgcagctggtgagaaatatcatacctcctctgtcttccacaaagcac
 **M**  E  N  T  L  Q  L  V  R  N  I  I  P  P  L  S  S  T  K  H 
aaagggcaagatggaagaataggcgtagttggaggctgtcaggagtacactggagcccca
 K  G  Q  D  G  R  I  G  V  V  G  G  C  Q  E  Y  T  G  A  P 
tattttgcagcaatctcagctctcaaagtgggcgcagacttgtcccacgtgttctgtgcc
 Y  F  A  A  I  S  A  L  K  V  G  A  D  L  S  H  V  F  C  A 
agtgcggccgcacctgtgattaaggcctacagcccggagctgatcgtccacccagttctt
 S  A  A  A  P  V  I  K  A  Y  S  P  E  L  I  V  H  P  V  L 
gacagccccaatgctgttcatgaggtggagaagtggctgccccggctgcatgctcttgtc
 D  S  P  N  A  V  H  E  V  E  K  W  L  P  R  L  H  A  L  V 
gtaggacctggcttgggtagagatgatgcgcttctcagaaatgtccagggcattttggaa
 V  G  P  G  L  G  R  D  D  A  L  L  R  N  V  Q  G  I  L  E 
gtgtcaaaggccagggacatccctgttgtcatcgacgcggatggcctgtggctggtcgct
 V  S  K  A  R  D  I  P  V  V  I  D  A  D  G  L  W  L  V  A 
cagcagccggccctcatccatggctaccggaaggctgtgctcactcccaaccacgtggag
 Q  Q  P  A  L  I  H  G  Y  R  K  A  V  L  T  P  N  H  V  E 
ttcagcagactgtatgacgctgtgctcagaggccctatggacagcgatgacagccatgga
 F  S  R  L  Y  D  A  V  L  R  G  P  M  D  S  D  D  S  H  G 
tctgtgctaagactcagccaagccctgggcaacgtgacggtggtccagaaaggagagcgc
 S  V  L  R  L  S  Q  A  L  G  N  V  T  V  V  Q  K  G  E  R 
gacatcctctccaacggccagcaggtgcttgtgtgcagccaggaaggcagcagccgcagg
 D  I  L  S  N  G  Q  Q  V  L  V  C  S  Q  E  G  S  S  R  R 
tgtggagggcaaggggacctcctgtcgggctccctgggcgtcctggtacactgggcgctc
 C  G  G  Q  G  D  L  L  S  G  S  L  G  V  L  V  H  W  A  L 
cttgctggaccacagaaaacaaatgggtccagccctctcctggtggccgcgtttggcgcc
 L  A  G  P  Q  K  T  N  G  S  S  P  L  L  V  A  A  F  G  A 
tgctctctcaccaggcagtgcaaccaccaagccttccagaagcacggtcgctccaccacc
 C  S  L  T  R  Q  C  N  H  Q  A  F  Q  K  H  G  R  S  T  T 
acctccgacatgatcgccgaggtgggggccgccttcagcaagctctttgaaaccgaccca
 T  S  D  M  I  A  E  V  G  A  A  F  S  K  L  F  E  T  D  P 
gctttcttgtacaaagtggttcgatcttacacgggatccgattacaaggatgacgacgat
 A  F  L  Y  K  V  V  R  S  Y  T  G  S  D  Y  K  D  D  D  D 
aagggcgcagattacaaggatgacgacgataaggcatga
 K  G  A  D  Y  K  D  D  D  D  K  A  -

**References cited in Supplementary Information**

Borna, N.N., Kishita, Y., Abe, J., Furukawa, T., Ogawa-Tominaga, M., Fushimi, T., Imai-Okazaki, A., Takeda, A., Ohtake, A., Murayama, K., Okazaki, Y., 2020. NAD(P)HX dehydratase protein-truncating mutations are associated with neurodevelopmental disorder exacerbated by acute illness. Brain 143(7), e54. <https://doi.org/10.1093/brain/awaa130>.

Drummond, K.J., Spiteri, M., Cain, S.A., Jones, J., Shaya, S., Topp, M., Lu, T., Tobler, R., Valkovic, A.L., Moore, Z., Fatunla, O.E., Kriel, J., Moffet, J.J.D., McAlpine, H., Rosier, M., Guan, H., Dimou, J., Schadewaldt, V., Roberts-Thomson, S., McArdle, D., Lui, E., Voelker-Albert, M., di Sanzo, S., Nijagal, B., Narayana, V.K., Mitchell, C.B., Vissers, J.H.A., Grimmond, S., Rosenthal, M.A., Palmer, L.M., Best, S.A., Freytag, S., Whittle, J.R., 2025. Perioperative IDH inhibition in treatment-naive IDH-mutant glioma: a pilot trial. Nat Med. <https://doi.org/10.1038/s41591-025-03884-4>.

Fiehn, O., Robertson, D., Griffin, J., van der Werf, M., Nikolau, B., Morrison, N., Sumner, L.W., Goodacre, R., Hardy, N.W., Taylor, C., Fostel, J., Kristal, B., Kaddurah-Daouk, R., Mendes, P., van Ommen, B., Lindon, J.C., Sansone, S.-A., 2007. The metabolomics standards initiative (MSI). Metabolomics 3(3), 175-178. <https://doi.org/10.1007/s11306-007-0070-6>.

Johnston, A.J., Hoogenraad, J., Dougan, D.A., Truscott, K.N., Yano, M., Mori, M., Hoogenraad, N.J., Ryan, M.T., 2002. Insertion and assembly of human tom7 into the preprotein translocase complex of the outer mitochondrial membrane. J Biol Chem 277(44), 42197-42204. <https://doi.org/10.1074/jbc.M205613200>.

Manor, J., Calame, D., Gijavanekar, C., Tran, A., Faith, J., Lalani, S.R., Mizerik, E., Parnes, M., Mehta, V.P., Lupski, J.R., Scagila, F., Elsea, S.H., 2022. Niacin therapy improves clinical outcome with normalization of metabolic abnormalities in a patient with NAXD deficiency. Brain.

Van Bergen, N.J., Gunanayagam, K., Bournazos, A.M., Walvekar, A.S., Warmoes, M.O., Semcesen, L.N., Lunke, S., Bommireddipalli, S., Sikora, T., Patraskaki, M., Jones, D.L., Garza, D., Sebire, D., Gooley, S., McLean, C.A., Naidoo, P., Rajasekaran, M., Stroud, D.A., Linster, C.L., Wallis, M., Cooper, S.T., Christodoulou, J., 2023. Severe NAD(P)HX Dehydratase (NAXD) Neurometabolic Syndrome May Present in Adulthood after Mild Head Trauma. Int J Mol Sci 24(4). <https://doi.org/10.3390/ijms24043582>.

Van Bergen, N.J., Guo, Y., Rankin, J., Paczia, N., Becker-Kettern, J., Kremer, L.S., Pyle, A., Conrotte, J.F., Ellaway, C., Procopis, P., Prelog, K., Homfray, T., Baptista, J., Baple, E., Wakeling, M., Massey, S., Kay, D.P., Shukla, A., Girisha, K.M., Lewis, L.E.S., Santra, S., Power, R., Daubeney, P., Montoya, J., Ruiz-Pesini, E., Kovacs-Nagy, R., Pritsch, M., Ahting, U., Thorburn, D.R., Prokisch, H., Taylor, R.W., Christodoulou, J., Linster, C.L., Ellard, S., Hakonarson, H., 2019. NAD(P)HX dehydratase (NAXD) deficiency: a novel neurodegenerative disorder exacerbated by febrile illnesses. Brain 142(1), 50-58. <https://doi.org/10.1093/brain/awy310>.

Zhou, J., Li, J., Stenton, S.L., Ren, X., Gong, S., Fang, F., Prokisch, H., 2019. NAD(P)HX dehydratase (NAXD) deficiency: a novel neurodegenerative disorder exacerbated by febrile illnesses. Brain. <https://doi.org/10.1093/brain/awz375>.
